# Benchmarking and optimisation of bait-capture metagenomics for sequencing of respiratory viruses at scale

**DOI:** 10.1186/s13073-026-01725-8

**Published:** 2026-07-30

**Authors:** Josef Wagner, Diana Rajan, Sarah Frances Field, Emma Betteridge, Katie Bellis, Marissa Knoll, Duncan Ng, Diego Teixeira, Beth Blane, Asha Akram, Catarina de Sousa, Joe Brennan, Dinesh Aggarwal, George MacIntyre-Cockett, Sandra E. Chaudron, Nicholas Grayson, Ben Hyatt, Andrew Wong, Anastasia Galvin, Ya-Lin Huang, David Jackson, Matthew Forbes, Frank Schwach, Andrea Frick-Kretschmer, Katerina Figueroa, Florent Lassalle, William Roberts-Sengier, Adrianne Lignes, Fernanda Novaes, Salma Fatima, Kevin Howe, Sara Stott, David Bonsall, Ewan M. Harrison

**Affiliations:** 1 Wellcome Sanger Institute, Wellcome Genome Campus, Hinxton, Cambridge, CB10 1SA UK; 2 Department of Medicine, University of Cambridge, Addenbrooke’s Hospital, Cambridge, CB2 0QQ UK; 3 Department of Infectious Disease, Imperial College London, London, W2 1PG UK; 4 Nuffield Department of Medicine, Centre for Human Genetics, Roosevelt Dr, Headington, Oxford, OX3 7BN UK; 5 Nuffield Department of Medicine, Pandemic Sciences Institute, University of Oxford, Old Road Campus, Oxford, OX3 7DQ UK

## Abstract

**Background:**

Sequencing respiratory virus genomes is essential for public health surveillance and research. Although shotgun metagenomics is pathogen agnostic, its sensitivity is limited by abundant off-target host nucleic acids. Hybridization bait capture overcomes this limitation by selectively enriching viral sequences prior to sequencing.

**Methods:**

We evaluated three respiratory viral bait capture workflows (veSEQ, RVI-seq, and Illumina) to compare their performance and assess their suitability for scalable respiratory virus sequencing. Synthetic RNA controls and clinical samples containing SARS-CoV-2, influenza A, influenza B, human parainfluenza virus, and respiratory syncytial virus (RSV) were analysed.

**Results:**

All three workflows demonstrated high efficiency, reproducibility and broadly comparable performance across respiratory viruses. Complete viral genomes were consistently recovered from samples containing 10,000 viral copies, while viral reads remained detectable at substantially lower viral loads, including approximately 100 copies. Workflow optimisation reduced reagent costs and enabled laboratory automation without compromising sequencing sensitivity.

**Conclusion:**

Hybridization bait capture provides an effective and scalable approach for respiratory virus genome sequencing. Cost reductions and automation can be implemented without compromising performance, supporting its use in routine genomic surveillance and public health preparedness.

**Supplementary Information:**

The online version contains supplementary material available at https://doi.org/10.1186/s13073-026-01725-8.

## Background

The biological and epidemiological understanding of viral pathogens has been transformed by whole genome sequencing [1–4]. ‘Real-time’ sequencing has helped shape the public health response to major outbreaks like Ebola [5–7], Zika [8–10], and most prominently during the COVID-19 pandemic, when sequencing of SARS-CoV-2 was carried out at previously unprecedented scale and in a global manner. This enabled real-time epidemiological surveillance and in combination with experimental data, a rapid genotype–phenotype characterisation of viral mutations and variants of concern [11–15].

The burden of viral respiratory infections, combined with the continual risks of emerging novel viruses highlights an urgent need for a global surveillance tool kit capable of detecting novel variants and species, and rapid scaling to high-throughput to support outbreak investigations and containment efforts. While amplicon-based methods are cost-effective, they are typically constrained to a single viral species and require pre-existing knowledge of specific genomes to design PCR primers. Shotgun metagenomics offers the advantage of sequencing all nucleic acids in a sample agnostic to the pathogen, enabling simultaneous detection of multiple species and discovery of novel pathogens, but this approach is hindered by the dominance of host and microbial DNA, which limits sensitivity and can inflate costs [16].

To overcome this limitation, viral bait capture (or hybridization capture) has emerged as a powerful method, selectively enriching for viral sequences in complex samples. Viral bait capture enables simultaneous detection of multiple viral species, including novel species with substantial divergence from probe designs (up to 20% divergence between probes and genome-fragments) and characterisation of co-infections from single samples, making it a versatile tool for viral genomics [17, 18]. Bait capture has been successfully applied across a range of pathogens, including bacteria, fungi, parasites, and viruses (for a review see [19]) with recent advances enabling the design of comprehensive viral panels that target a broad spectrum of viruses. For example, a cost-effective HIV probe-based NGS method (veSEQ) was successfully used to recover HIV genomes from 91% of samples with a viral load above 1,000 copies/ml [20]. The same veSEQ protocol was modified during the COVID-19 pandemic and used to analyse 1,313 clinical samples, focusing on SARS-CoV-2 within-host diversity and transmission [21]. In 2020, a study compared sequencing methods for SARS-CoV-2 and found that bait capture enrichment provided results similar to amplification methods [13]. Despite its ability to enhance viral read recovery up to 100-fold, viral bait capture has not yet been adapted for very high-throughput automated workflows [22].

A number of different viral bait capture methods have been developed and used [23, 24], however no direct comparisons have been made to determine the strengths and weakness of different viral bait capture methods for respiratory viruses. To address this gap, we sought to evaluate three different viral bait capture workflows, i.e., veSEQ workflow, RVI-seq workflows, and Illumina workflow to identify relative advantages and to assess their potential for scalable respiratory virus sequencing. We processed synthetic RNA and clinical samples, including SARS-CoV-2, influenza A and B, human parainfluenza virus, and respiratory syncytial virus (RSV). Overall, our work demonstrates that all three methods performed comparably well. Overall, our results demonstrate that the broad effectiveness of viral bait capture methods for respiratory viruses and show cost reductions can be made without impacting performance.

## Methods

### Sample and nucleic acid extraction

**Fig. 1:**
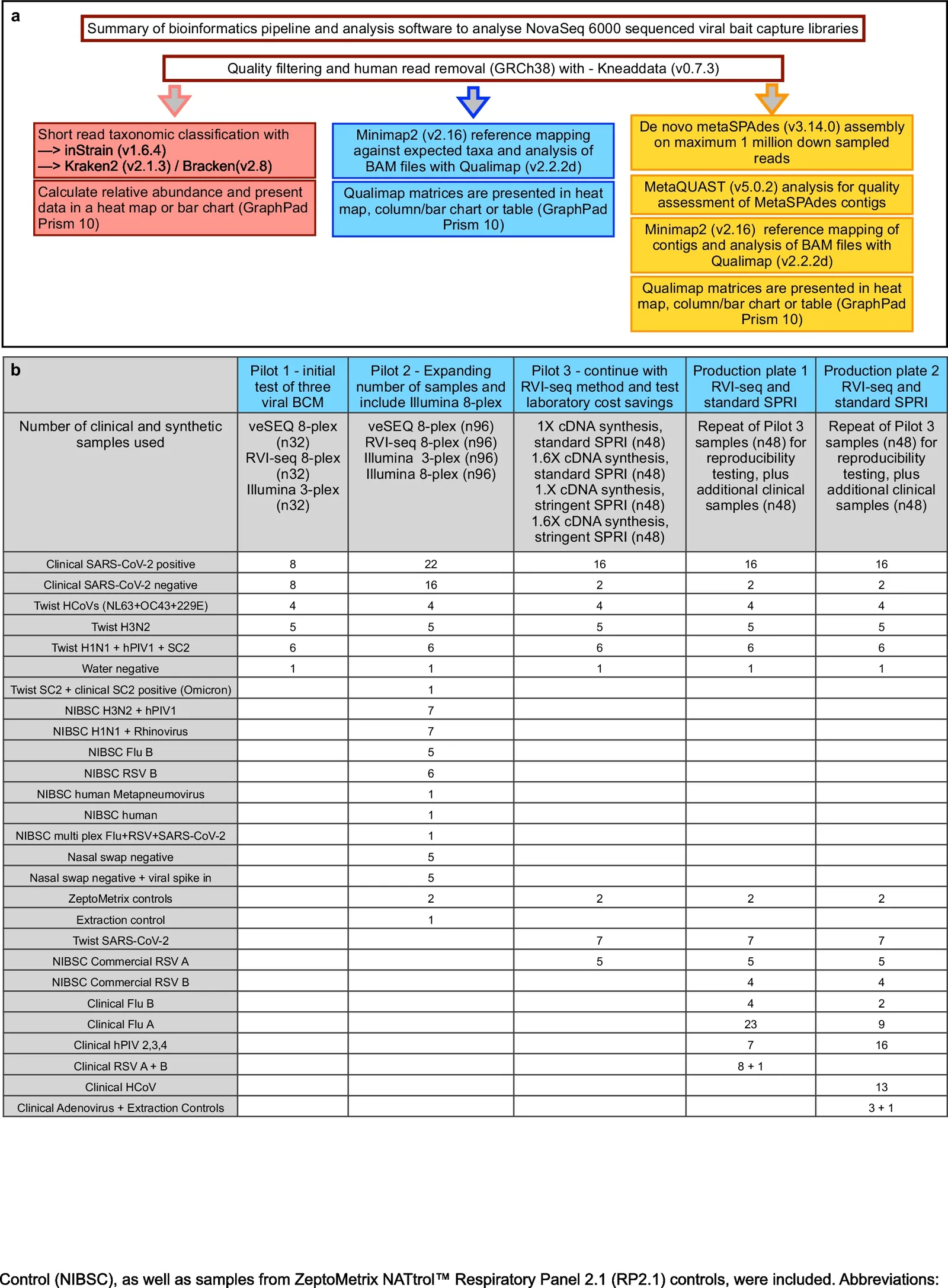
Flowchart outlining bioinformatics pipeline and samples used for validating three viral bait capture methods (BCM). **a** The bioinformatics pipeline includes programs for short read taxonomic classification, reference mapping, de novo assembly, BAM file and contigs analysis tools. **b** Samples include clinical samples of SARS-CoV-2 (SC2), influenza A (H1N1 and H3N2), influenza B, human parainfluenza viruses (hPIV 2, 3, and 4), respiratory syncytial viruses (RSV A and B), non-SARS human coronaviruses (hCoV), and adenovirus. Additionally, synthetic RNA from Twist and reference RNA from the National Institute for Biological Standards and Control (NIBSC), as well as samples from ZeptoMetrix NATtrol™ Respiratory Panel 2.1 (RP2.1) controls, were included. Abbreviations: SPRI = Solid-Phase Reversible Immobilisation

Details of the sample types, sample names, and sequence file IDs used in Pilot Studies 1–3 and Production Plates 1–2 is provided in Additional file 1: Table S1. Sample sizes for each pilot study and production plate are shown in Fig. 1.

Clinical samples were provided as nucleic acid extracts. Extraction occurred in multiple UK diagnostic laboratories using either the PerkinElmer Chemagic 360 with proprietary kits or the Thermo Fisher KingFisher system (with or without Thermo Fisher Amplitude) and MagMax Viral/Pathogen kits. Both methods included MS2 phage as an internal control. After diagnostic testing, these samples were provided for our use and stored at or below −20 °C.

Commercial controls from Amplirun were reconstituted according to the manufacturer’s instructions. On-site extractions of commercial controls from NIBSC [25] and Zeptometrix [26] were performed using a KingFisher Apex with the MagMax Viral/Pathogen Ultra Nucleic Acid Isolation Kit (A42356), following the manufacturer’s recommendations. The enzyme and buffer mix were prepared before sample addition, rather than adding the enzyme directly to the buffer/sample mix. Synthetic viral RNA controls from Twist Bioscience were spiked into reactions at various input amounts and combinations, using a background of 50 ng Universal Human Reference RNA (Invitrogen) per reaction.

NIBSC stands for National Institute for Biological Standards and Control [25]. It is a UK-based organization that develops and distributes biological reference materials and standards for healthcare and research.

Controls used in this study were:

- Influenza A (H1N1) Panel 20/180; Influenza A (H3N2) Panel 20/182; Influenza B Panel 20/184, Human Adenovirus DNA for nucleic acid amplification techniques (1st WHO international Standard) 16/324; Human Metapneumovirus Working Reagent for Nucleic Acid Amplification Testing 08/320; Parainfluenza virus serotype 1 for Nucleic Acid Amplification Techniques 08/176; Parainfluenza virus serotype 2 for Nucleic Acid Amplification Techniques 08/178; Human Rhinovirus Genogroup A Working Reagent for Nucleic Acid Amplification Testing. Type 39 08/324.
- ZeptoMetrix NATtrol™ Respiratory Panel 2.1 (RP2.1) [26] Controls are commonly used in molecular diagnostics and assay development for detecting respiratory pathogens, including viruses like SARS-CoV-2, influenza, and RSV (Catalog Number: NATRPC2.1-BIO).
- Synthetic RNA controls were obtained from Twist Bioscience [27] (Twist Bioscience, South San Francisco, CA, USA).

Samples were residual total nucleic acid from COVID-19 diagnostic testing nasal/throat swabs—either positive or negative SARS-CoV-2 samples (*n =* 16; qPCR confirmed), Twist synthetic RNAs representing: seasonal human coronaviruses: OC43, NL63, 229E; influenza A: H3N2 and H1N1 dilution series (10^6 –^ 10^2^); Human parainfluenza virus 1 (HPIV-1), and SARS-CoV-2, and mixes of the latter three). For Illumina we followed the manufacturer’s instructions and spiked-in MS2 phage into samples as an internal control. Samples were pooled at 3-plex for the pull-down (bait capture) using the Respiratory Virus Oligo Panel (RVOP) and Respiratory Pathogen ID/AMR Enrichment Panel (RPIP) combined in a 1:1 ratio. The veSEQ and RVI-seq method samples were pooled at 8-plex for pull-down with the Twist Bioscience Respiratory Virus Research Panel (for detailed information see Material and Methods).

### Viral bait capture library preparation and sequencing

#### Pilot 1

In all methods, a fixed volume of 8ul sample (total nucleic acid extract) is used as input into the workflow, regardless of nucleic acid concentration or Ct value.

The Illumina workflow was performed using the Respiratory Virus Enrichment kit, according to manufacturer’s recommendations. The Respiratory Pathogen ID/AMR Enrichment Panel (Illumina) was combined with the Respiratory Virus Oligo Panel v2 (Illumina) in a 1:1 ratio. 4.8 × 10^6^ copies of MS2 RNA (Roche) were spiked into all samples. 200 ng of each library where available were pooled at 3-plex or 8-plex prior to bait capture. Plex refers to the number of different samples which can be combined in one bait capture reaction.

A modified version of the veSEQ workflow [28] was performed:

- Libraries were generated with the SMARTer Stranded Total RNA-Seq Kit v2 Pico Input Mammalian according to manufacturer’s recommendations (Takara Bio USA), following the no fragmentation option and skipping the ribodepletion step.
- 200 ng of each library where available was pooled at 8-plex prior to bait capture
- Bait capture was performed following the Twist Target Enrichment Standard Hybridization v1 protocol (Twist Bioscience) and Respiratory Virus Research Panel (Twist Bioscience)
- Following the final post-capture wash, streptavidin beads were resuspended in 25ul nuclease-free water and 22.5ul was taken into PCR
- Post capture PCR (12 cycles) was performed with KAPA HiFi HotStart ReadyMix (Roche)

RVI-seq workflow

- cDNA synthesis was performed using the NEBNExt Ultra II Directional RNA library prep kit (New England Biolabs), following the protocol version without fragmentation. Reagent volumes are scaled to 1.6X to accommodate 8ul sample input. Samples are eluted in 1XTE.
- Library preparation was performed using the NEBNext Ultra II FS DNA Library Prep Kit for Illumina (New England Biolabs), with a 15 min fragmentation incubation.
- Library PCR (12 cycles) was performed with KAPA HiFi HotStart ReadyMix (Roche)
- 200 ng of each library, where available, was pooled at 8-plex prior to bait capture.
- Bait capture was performed following the Twist Target Enrichment Standard Hybridization v1 protocol (Twist Bioscience) and Respiratory Virus Research Panel (Twist Bioscience)
- Following the final post-capture wash, streptavidin beads were resuspended in 25ul water and 22.5ul was taken into PCR
- Post capture PCR (12 cycles) was performed with KAPA HiFi HotStart ReadyMix (Roche)
- The detailed protocol for the RVI-seq method is available on protocols.io [29]

These are the reference links for the TWIST and Illumina websites which list the targets for each enrichment panel/method:

TWIST: [30, 31]

Illumina [32–34]:

Pilot 1: combined RPIP and RVoP v2 probes 1:1

Pilot 2: RVoP v2 probes only.

#### Pilot 2

The Illumina workflow was performed using the Respiratory Virus Enrichment kit [33], according to manufacturer’s recommendations. Only the Respiratory Virus Oligo Panel v2 was used, and MS2 was not spiked into samples. Libraries were pooled at both 3-plex and 8-plex prior to bait capture.

The veSEQ workflow was performed as described for pilot 1.

The RVI-seq workflow was performed as described for pilot 1, except the fragmentation incubation time in library preparation was reduced from 15 to 12 min.

#### Pilot 3

The RVI-seq workflow was performed as described for pilot 2, except the first strand synthesis incubation time was increased from 15 to 50 min.

Forty-eight samples were processed in replicate, comparing cDNA synthesis volumes run at 1.6X vs 1X scale, whilst maintaining sample input volume at 8ul.

The final pool was also subjected to a further SPRI cleanup (0.8X) and sequenced separately (referred to as the ‘stringent’ SPRI condition in the main text).

### Sequencing

Equimolar pools were sequenced on the Illumina NovaSeq 6000, on SP flow cells with the XP workflow, 150PE at 96 samples per lane. For the pilot 1 study only, the combined libraries of all 96 samples were sequenced in duplicate.

## Bioinformatics and data analysis

These bioinformatic analyses steps were collectively executed by running the two Nextflow pipelines available at the addresses listed below, except for step 3.7: [35] https://github.com/sanger-pathogens/rvi-viral-metagenomics-pipeline. Using code version v1.4.5 for the abundance estimation and assembly tasks (steps 3.1, 3.2, 3.3, 3.6 and 3.8: [36] https://github.com/sanger-pathogens/rvi-viral-lens (steps 3.4, 3.5, 3.9). Quality assessment of de novo assembled contigs (step 3.7) was achieved using custom code in shown in 3.7.

### Sequence quality filtering with Kneaddata (v 0.7.3) [37]

Kneaddata was used to remove low quality reads and human reads. Two different sequencer-source options were used depending on whether the samples were prepared with the veSEQ and RVI-seq method or with the Illumina method. The human reference genome GRCh38 was obtained from [38]. http://hgdownload.cse.ucsc.edu/goldenPath/hg38/bigZips/. Script for the two different sequencer-source options.

with NexteraPE sequencer-source for Illumina BCM

Kneaddata -i1 ${MYINPUT}_1.fastq.gz -i2 ${MYINPUT}_2.fastq.gz –run-fastqc-start –run-fastqc-end -t 32 -db Homo_sapiens -o Results.0.12.0.Illumina/${MYINPUT} –sequencer-source NexteraPE –trimmomatic-options "ILLUMINACLIP:/data/pam/software/solexa/solexa-with-nextseqPR-adapters.fasta:2:10:7:1 LEADING:3 TRAILING:3 CROP:150 SLIDINGWINDOW:4:20 MINLEN:100".

with TruSeq3 sequencer-source for veSEQ and RVI-seq BCMs

Kneaddata -i1 ${MYINPUT}_1.fastq.gz -i2 ${MYINPUT}_2.fastq.gz –run-fastqc-start –run-fastqc-end -t 16 -db homo_sapiens -o Results/${MYINPUT} –sequencer-source TruSeq3 –trimmomatic-options "ILLUMINACLIP:/data/pam/software/solexa/solexa-with-nextseqPR-adapters.fasta:2:10:7:1 LEADING:3 TRAILING:3 CROP:150 SLIDINGWINDOW:4:20 MINLEN:100".

Kraken2/Bracken analysis [39–41]

This analysis made use of three tools: Kraken2 v2.1.3, Bracken v2.8 and Krakentools v1.2.

kraken2 –db database.k2db \

--hreads 4 \
--output sample_id_kraken_report.tsv \
--use-names \
--report sample_id_kraken_sample_report.tsv \
--report-zero-counts \
--paired reads_1.fastq.gz reads_2.fastq.gz.
  Then Bracken was used to provide an estimate abundance at the species level:
  est_abundance.py -i sample_id_kraken_sample_report.tsv \
  - k database150mers.kmer_distrib \
  - o sample_id.bracken \
  - l "S" \
  - t 1

where database150mers.kmer_distrib is a Bracken database generated from the Kraken database using steps described on [42] https://ccb.jhu.edu/software/bracken/index.shtml?t=manual#step1. To effectively automate the process, we used the manual steps 1a, 1b, 1c. However, the same file would be generated if you used the single step 1 described there:

- bracken-build -d database.k2db -t 10 -k 35 -l 150 -x kraken2

The Bracken output was converted into a more convenient format for downstream processing using krakentools (v1.2). In particular, the reports were converted to a tabular format:

- kreport2mpa.py -r sample_id.bracken -o tabulated_report.tsv –intermediate-ranks –display-header

The tabulated reports were then combined using:

- combine_mpa.py -i all_tabulated_reports/* -o summary_report.tsv.

This analysis was repeated for 3 different Kraken2 databases. In the end we settled on PlusPFP 3/14/2023 [43]. (https://genome-idx.s3.amazonaws.com/kraken/k2_pluspfp_20230314.tar.gz) available on Ben Langmead's index site: [44] https://benlangmead.github.io/aws-indexes/k2.

##  inStrain analysis

For high-resolution reference-based analyses, including influenza strain- and segment-level comparisons, we used inStrain, as Kraken does not reliably distinguish between closely related viral strains or segmented genomes. InStrain enables mapping-based genome comparisons at nucleotide-level resolution, allowing more detailed evaluation of capture performance. To ensure consistency between approaches, we performed parallel Kraken analyses and confirmed concordance at shared taxonomic levels.

For the inStrain [45] analysis, the NCBI Viral RefSeq database (viral.1.1.genomic.fna.gz 2023–09-09) was used. All reads were first mapped using bowtie2 (v2.5.1) and sorted with samtools (v1.17):

- bowtie2 -p 4 -x viral.1.1.genomic_sorted.bt2 −1 reads_1.fastq.gz −2 reads_2.fastq.gz | samtools sort -@ 4 -o sample_id.sorted.bam

where the bowtie2 index files were generated with:

- bowtie2-build –large-index viral.1.1.genomic_sorted.fasta viral.1.1.genomic_sorted

InStrain (v1.6.4) was then run on the mapped reads:

- inStrain profile sample_id.sorted.bam viral.1.1.genomic sorted.fasta -o instrain_output -p 8 -s viral.1.1.genomic.stb –database_mode –skip_plot_generation

where viral.1.1.genomic.stb was generated using bash commands.

Per-sample inStrain output was then combined using bash scripts into a single summary abundance table.

For the taxonomic classification of high quality (HQ) paired-end short reads, we optimised inStrain by comparing three different option settings and then compared the best inStrain option with Kraken2/Bracken analysis. This optimisation method was first tested on sequence data from the ZeptoMetrix Respiratory Panel 2.1 (RP2.1) controls and then on HQ paired-end short reads (downsampled to a maximum of 1 million reads) from the Pilot 2 viral bait capture experiment. The ZeptoMetrix Respiratory Panel was sequenced together with the Pilot 2 samples in the same library.

The three different inStrain options were:

- option 1 "with database mode, min genome coverage 1, min read average nucleotide identity (ani) of (0.92) and skip mm profiling"
- option 2 "no database mode, min genome coverage 0, min read ani of 0.9"
- option 3 (default) "no database mode, min genome coverage 0, min read ani of 0.95"

### Mapping of HQ paired end short reads

Mapping of HQ paired-end short reads was performed on two different minimum length filtered datasets, comparing mapping and de novo assembly results between 70 and 100 bp minimum length datasets. Mapping was performed with minimap2 (v 2.16) [46, 47] using the following command script, together with samtools view and samtools sort (v 1.9) [48].

minimap2 -x sr -a reference.fasta ${MYINPUT}_1.fastq.gz ${MYINPUT}_2.fastq.gz | samtools view -F 4 -h −1 -S -b | samtools sort -o ${MYINPUT}_outputfile.sorted.bam.

### Quality assessment of mapping

Sequence mapping was assessed with Qualimap (v 2.2.2d) [49] using the following command line: qualimap –java-mem-size = 10000 M bamqc -bam $MYINPUT.

A list file containing all the mapped bam files has been used as “MYINPUT”.

### De novo assembly

De novo assembly using HQ paired end short reads was performed with “–meta” (metaSPAdes) [50] option from SPAdes program [51] (v 3.14.0) using the following command script:

- spades.py –meta –threads 16 −1 ${MYINPUT}_1_kneaddata.trimmed.1.fastq.gz −2 ${MYINPUT}_1_kneaddata.trimmed.2.fastq.gz -o ${MYINPUT}_3.14.0.meta.spades.

A list file containing fastq files has been used as “MYINPUT”.

### Quality assessment of de novo assembled contigs

#### MetaSPAdes assembly quality scoring with MetaQUAST (v5.0.2) [52]

MetaSPAdes-assembled contigs were quality scored using MetaQUAST (version 5.0.2). For input, multiple scaffolds.fasta files were provided using a wildcard option, as shown in the example below, along with several reference datasets. For gene searches, the **–**glimmer option was utilized to enhance the identification of coding regions.

Example scaffold.fasta filename:

47096_2#96_3.14.0.meta.spades_scaffolds.fasta.

metaquast.py*.meta.spades_*.fastathreads64m500glimmerrHuman_coronavirus_229E.fasta,Human_coronavirus_NL63.fasta,Human_coronavirus_OC43.fasta,Human_parainfluenca_virus1.fasta,Influenza_A_H1N1_segment1.fasta,Influenza_A_H1N1_segment2.fasta,Influenza_A_H1N1_segment3.fasta,Influenza_A_H1N1_segment4.fasta,Influenza_A_H1N1_segment5.fasta,Influenza_A_H1N1_segment6.fasta,Influenza_A_H1N1_segment7.fasta,Influenza_A_H1N1_segment8.fasta,Influenza_A_H3N2_segment1.fasta,Influenza_A_H3N2_segment2.fasta,Influenza_A_H3N2_segment3.fasta,Influenza_A_H3N2_segment4.fasta,Influenza_A_H3N2_segment5.fasta,Influenza_A_H3N2_segment6.fasta,Influenza_A_H3N2_segment7.fasta,Influenza_A_H3N2_segment8.fasta,Wuhan_Hu_NC045512.2.fasta.

### Downsampling to 1 million forward and reverse reads

We use seqtk to perform random read sampling using a fixed random seed for replicability. Subsampling upstream of metaSPAdes assembly was caping the paired-end reads sets to a size of 2 × 500 k reads. There was another upstream subsampling happening upstream of preprocessing to cap the total number of read considered for both assembly and abundance estimation with Kraken2/Bracken and inStrain to 2 × 10 M reads.

### Heatmap generation and data plotting

Kraken2 and inStrain absolute abundances were converted to relative abundances using Microsoft Excel for Mac (v 16.82), and relative abundances of viral taxa were plotted in a heatmap using GraphPad Prism (v 10.2.0). GraphPad Prism was also used for all other plots, including bar plots, column plots and scatterplot plots, with appropriate error bars included in the plots.

### Statistical analysis

Statistical analyses were performed using GraphPad Prism (v10.2.0). Data were assessed for normality using the D'Agostino-Pearson omnibus test, Anderson–Darling test, and Shapiro–Wilk test. Since all datasets deviated from a normal (Gaussian) distribution, non-parametric tests were used for comparisons. For unpaired two-group comparisons, the Mann–Whitney U test was applied. For comparisons involving three or more unpaired groups, the Kruskal–Wallis test followed by Dunn’s multiple comparisons test was used. For paired comparisons across three or more groups, Friedman’s test with multiple comparisons was performed. Statistical significance was set at *p* < 0.05 (95% confidence level).

inStrain abundance data and minimap2 mapping data are presented as heat maps, with each cell indicating the relative inStrain abundance or the percentage of the genome covered in reference-based mapping.

### Turn Around Time (TAT)

Processing of one 96-well plate takes approximately 5 days, followed by 1 day for sequencing. Data processing and review require an additional 1–2 days.

## Results

### Pilot comparison of the three viral bait capture methods

We tested three different respiratory virus bait capture (hybridization capture**)** methods (henceforth: viral BCMs) to determine which method should be taken forward before use at scale within a centralised facility. The three methods tested were: firstly, the commercially available – Illumina Respiratory Virus Enrichment Kit [33] (henceforth: ‘Illumina’); secondly, the veSEQ method developed at the University of Oxford and previously used for sequencing of HIV and SARS-CoV-2 [28], and thirdly, we combined an in-house RNA-seq library preparation workflow with bait capture (henceforth: RVI-seq). The advantage of this latter protocol was that significant work had already been carried out to optimise reaction conditions and automate library preparation. RIV-seq and veSEQ methods differ in the library preparation but used a similar capture.

For the first pilot study, we sequenced 32 samples with each method, including one water negative control (Fig. 1 and Additional file 1: Table S1).

There was no significant difference in the number of raw read counts in the two duplicated sequencing lanes across all three methods (Mann–Whitney two-tailed *P =* 0.6124; Additional file 2: Fig. S1). Kraken classification indicated relative cellular vs. viral read proportions of 51% (52 million reads; ‘m’) and 49% (50 m) for the RVI-seq method; 31% (34.5 m) and 69% (75.3 m) for the veSEQ method; and 25% (23.9 m) and 75% (71.3 m) for the Illumina method (Additional file 3: Fig. S2). The proportion of viral reads for the Illumina method without the MS2 phage was 46%. Therefore, 29% of spiked MS2 phage in the Illumina method have contributed to the higher number of viruses detected (Additional file 4: Fig. S3). In contrast, the lower number of viruses observed with the RVI-seq method is likely a consequence of shorter fragment sizes generated during library preparation (Additional file 5: Fig. S4), a large proportion of which were removed during the initial Kneaddata filtering step, which retained only sequences ≥ 100 bp. As a result, following Pilot 1, we adjusted the minimum length threshold in the Kneaddata quality filtering step to 70 bp to reduce the loss of RVI-seq sequences and decreased the amount of MS2 spike in the Illumina method.

**Fig. 2:**
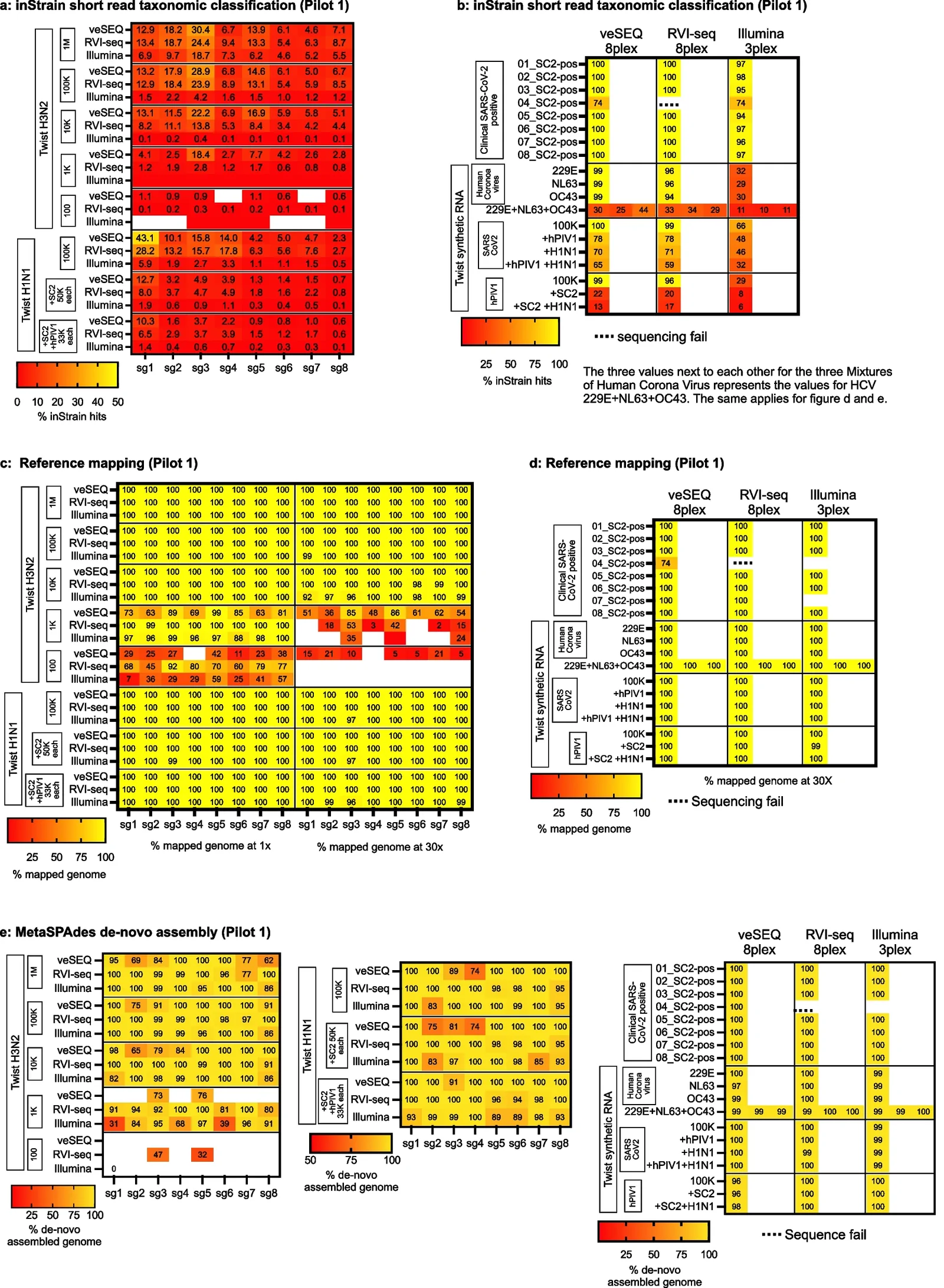
Comparisons of viral bait capture methods in Pilot 1 samples. High-quality paired-end reads (≥ 70 bp) were analysed using inStrain against the NCBI viral reference database (build 2023–05–09). **a** Relative inStrain abundances across eight segments of synthetic H3N2 (dilution series), H1N1 alone and in combination with SARS-CoV-2 (SC2) and human parainfluenza virus 1 (hPIV1), shown as a heat map. **b** Relative inStrain abundances for eight clinical SC2 samples, synthetic human coronaviruses (229E, NL63 and OC43), SC2 alone or in combination with hPIV1 and H1N1, and hPIV1 alone or in combination with SC2 and H1N1. Cells in the heat map with no value indicate abundances below 0.1%. **c** Reference mapping of the same reads to H3N2 and H1N1 reference genomes using minimap2 at 1 × and 30 × coverage. **d** Reference mapping at 30 × coverage for clinical SC2 and additional synthetic combinations. **e** Genome fraction assembled by MetaSPAdes de novo assembly for the same samples. Sample details are provided in Extended Data Table 1. Abbreviations: 1 M, 100 K, 10 K, 1 K and 100 refer to the RNA copy number in millions, thousands and hundreds, respectively

All three methods were similarly efficient at generating reads for the different synthetic viral RNAs and clinical SARS-CoV-2 samples (Fig. 2a). Lower efficiencies were noted with Illumina for synthetic human coronaviruses, SARS-CoV-2, and their mixtures, as well as for hPIV1 and their mixtures, compared to the other two methods in pilot 1 (Fig. 2b). One clinical SARS-CoV-2 sample processed with the RVI-seq method failed sequencing, but the RVI-seq method performed slightly better on the 100-copy Twist H3N2 sample. Reference mapping showed near full-length sequences at 30 × for H3N2 at 10,000 copies across all workflows, with context-dependent variation at 1,000 and 100 copies (Fig. 2c). RVI-seq yielded the most segments > 80% coverage at 1 × for 1 k copies, whereas veSEQ gave greater 30 × coverage at 1 k and 100 copies. Reference mapping for clinical SARS-CoV-2 samples was comparable between the veSEQ and RVI-seq methods, whereas two samples failed using the Illumina method (Fig. 2d). Illumina libraries contained high proportions of MS2 reads in 19/31 samples (> 50%, up to 100%), likely reducing target viral reads (Additional file 3: Fig. S2 and Additional file 3: Fig. S3), particularly for shorter influenza segments and non-SARS-CoV-2 positives. This likely contributed to the lower number of viral reads for Illumina samples. Analysis of the de novo assembly data revealed no major differences between the three viral BCMs overall (Fig. 2e), though for the individual H3N2 influenza segments, RVI-seq generated the largest contiguous assemblies from the 1 k copy number samples and partial fragment from 100 copy number samples that were not detected by the other two methods. Additional de novo assembly metrics with results are presented in the Additional file 6: Supplementary Result Section.

### Comparison across a broader range of samples

As the pilot showed no clear advantage with any BCMs we expanded the evaluation to 96 samples per workflow (Fig. 1 and Additional file 1: Table S1). To standardise Illumina with the other workflows, we removed the MS2 spike-in, used only the Respiratory Virus Oligo Panel (RVOP), for the hybridisation step, and tested both 3-plex (manufacturer recommended) and 8-plex pooling for the pull-downs. This enabled direct comparison with the veSEQ and RVI-seq methods (both 8-plex) and assessment of any sensitivity trade-off caused by increasing the number of indexed libraries combined for the pull-down step. Samples included: synthetic viral RNAs as used in pilot 1, clinical SARS-CoV-2 positive samples (qPCR Ct values: 11.6 to 25), a ZeptoMetrix control panel ^24^ containing a range of respiratory pathogens (NATtrol™ Respiratory Panel 2.1), UK National Institute for Biological Standards and Control (NIBSC), standards for a range of respiratory viruses (note for NIBSC control the copy numbers expressed are pre-extraction so will vary – see Methods for further details), and nasal swabs from asymptomatic volunteers that were spiked with a range of respiratory viruses and relevant controls.

As a first optimisation step, we analysed the differences between using a minimum length of 100 bp HQ reads, as used in pilot 1, and a minimum length of 70 bp HQ reads (used in Pilot 2 and subsequent analysis). Based on our findings, we reduced the minimum read size to 70 bp to ensure more reads generated by the RVI-seq method with the smaller insert size were included in the analysis (Additional file 5: Fig. S4).

**Fig. 3:**
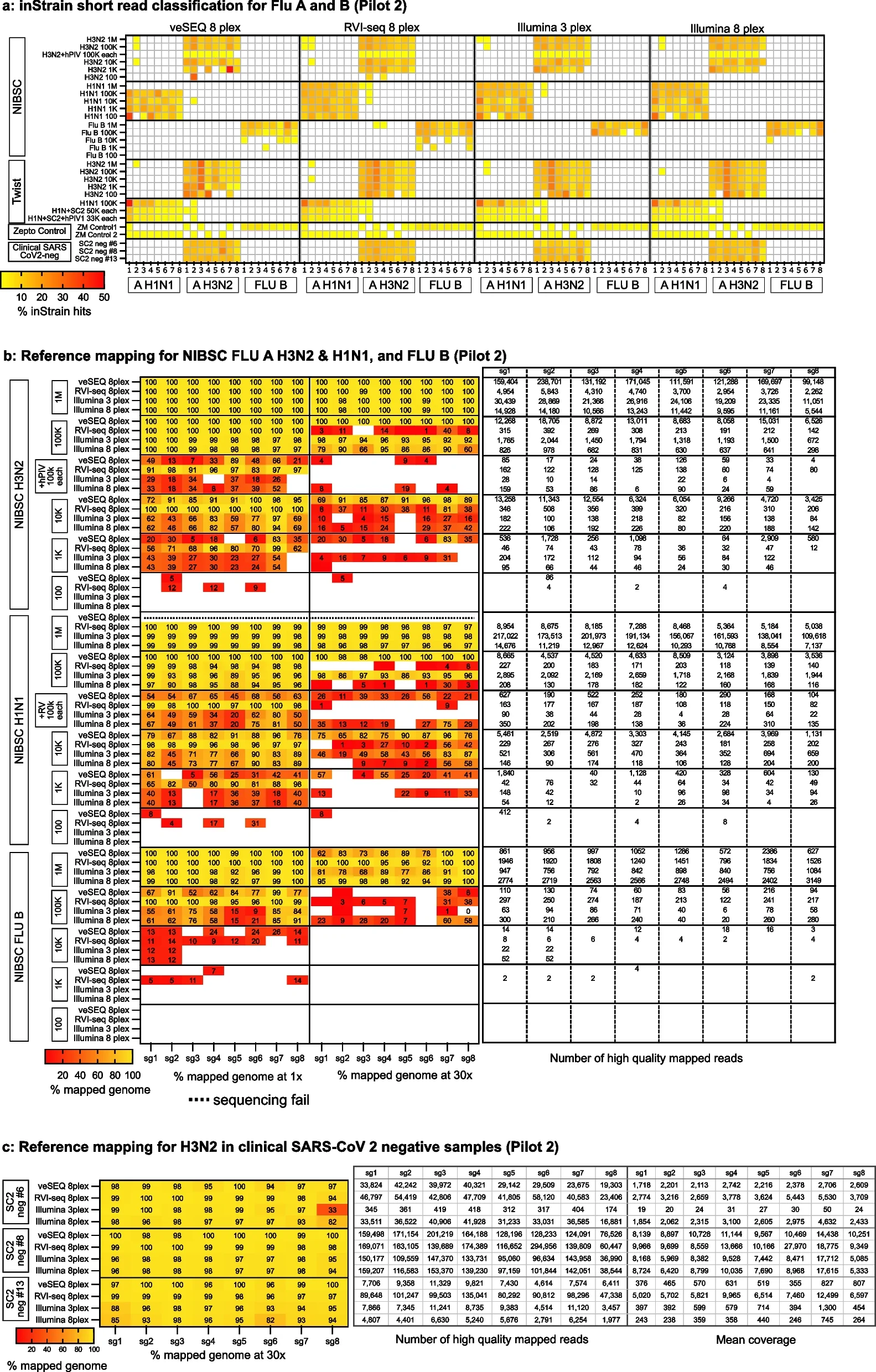
Short-read classification and reference mapping of influenza A and B samples in Pilot 2. High-quality paired-end reads (≥ 70 bp) were taxonomically classified using inStrain with the NCBI viral reference database (build 2023–05–09). **a** Relative inStrain abundances across the eight genome segments for the NIBSC and Twist influenza A and B dilution series in the presence or absence of hPIV1 and/or SARS-CoV-2 (SC2) across viral bait capture methods. **b** Reference mapping of the NIBSC samples using Minimap2 at 1 × and 30 × coverage alongside the number of high-quality mapped reads per segment (shown in the adjacent table). **c** Reference mapping results for three SC2-negative samples that were identified by inStrain as positive for H3N2. Abbreviations: NIBSC: National Institute for Biological Standards and Control; Twist: synthetic RNA (Twist Bioscience); SC2: SARS-CoV-2; hPIV1: human parainfluenza virus 1

**Fig. 4:**
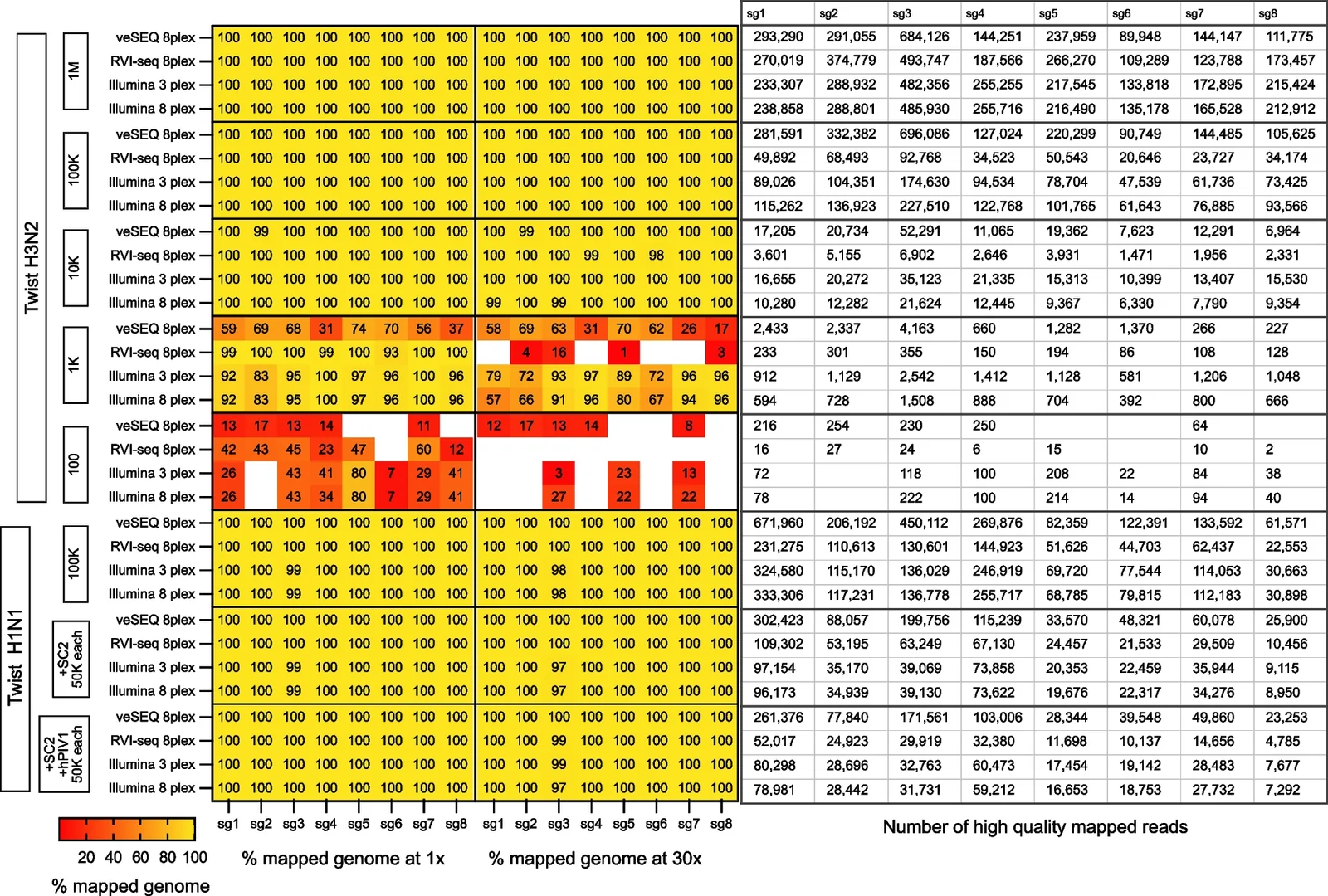
Mapping of H3N2 and H1N1, and comparison of BCMs in Pilot 2. **a** High-quality paired-end reads (≥ 70 bp) were reference-mapped for the Twist H3N2 dilution series and H1N1, either alone or in combination with SARS-CoV-2 and hPIV1. The reference mapping results are presented at 1 × and 30 × depths alongside the number of high-quality mapped reads per segment, as shown in the adjacent table. **b** Correlation analysis of the percentage of mapped genomes, the number of mapped reads and the mean coverage with qPCR Ct values for 18 clinical SARS-CoV-2 samples. Abbreviations: Twist: synthetic RNA control (Twist Bioscience); hPIV1: human parainfluenza virus 1

Using a range of serially diluted (10^6 –^ 10^2^) influenza A and B (either synthetic RNA (Twist Bioscience) or purified control samples (NIBSC), we compared the sensitivity of the three methods. Short read classification (inStrain) for both the H3N2 NIBSC and Twist serial dilutions detected all eight segments at levels down to 10 k copies (Fig. 3a) across workflows, with the RVI-seq method generating the most segments at 1 k and 100 copies. Similarly, H1N1 dilution series demonstrated comparable detection levels across veSEQ 8-plex, RVI-seq 8-plex, and Illumina 3 and 8- plex (Fig. 3a). Reference mapping was similar across the three BCMs for NIBSC H3N2 and H1N1 dilution series, although some differences were observed at low copy numbers (Fig. 3b). For example, RVI-seq generated more segments with small numbers of reads as 1 × depth, whereas veSEQ outperformed the other methods at 30 × depth. In contrast, reference mapping revealed less complete genome coverage for influenza B than was observed for influenza A overall, with RVI-seq and Illumina 8-plex BCMs showing better performance at 30 × depth. For the H3N2 clinical samples, all viral BCMs produced complete coverage at high read depth, except Illumina 3-plex showed a single segment-8 dropout (Fig. 3c). For Twist H3N2, performance assessed by reference mapping was highly consistent across all BCMs down to 10 k copies. At lower copy numbers, Illumina-based methods performed marginally better than both the veSEQ and RVI-seq methods at the 1 k copy level, especially at 30 × depth (Fig. 4). Reference mapping for Twist H1N1, alone or in combination with SARS-CoV-2 and hPIV1, was highly successful across all BCMs (Fig. 4).

**Fig. 5:**
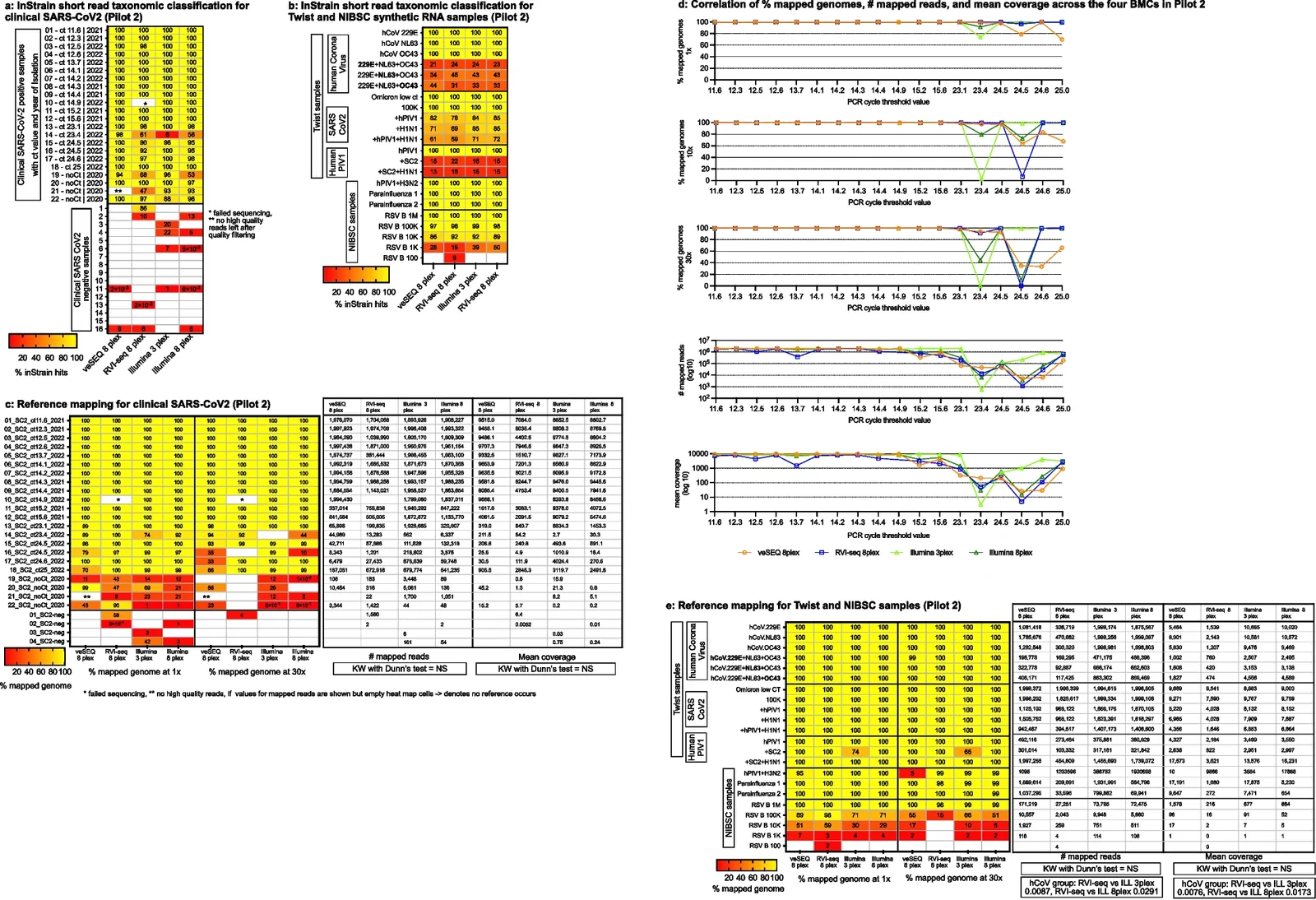
Short-read classification and reference mapping for SARS-CoV-2, human coronaviruses, human parainfluenza virus type 1 (hPIV1) and respiratory syncytial virus B (RSV B) (Pilot 2). High-quality paired-end reads (≥ 70 bp) were taxonomically classified using inStrain with the NCBI viral reference database (build 2023–05–09). **a** and **b** Relative inStrain hit abundances for clinical SARS-CoV-2-positive and -negative samples, Twist synthetic SARS-CoV-2 and human coronaviruses (229E, NL63 and OC43), hPIV1 alone and in mixtures (including with H1N1), NIBSC hPIV1 and hPIV2, and RSV B dilution series across viral bait capture methods (BCMs). In panel b, the strain name in bold corresponds to the value shown in the heat map. **c** Reference mapping of the same clinical SARS-CoV-2 samples was performed using Minimap2 at 1 × and 30 × coverage. The adjacent table shows the high-quality mapped read counts and mean coverage. In the mixed human coronavirus sample, the strain name in bold corresponds to the value shown in the heat map. **d** Reference mapping results for the same Twist and NIBSC samples at 1 × and 30 × coverage, with the corresponding mapping metrics shown. Statistical differences in the number of mapped reads and mean coverage across BCMs were tested using the Kruskal–Wallis test with Dunn’s post hoc test. Tests were performed across all samples and within individual sample groups. Significant pairwise differences are reported where applicable. Mapping metrics were generated using Qualimap v2.3, and heat maps and statistical analyses were performed in GraphPad Prism 10 (v10.3.1, macOS). Abbreviations: NIBSC: National Institute for Biological Standards and Control; Twist: synthetic RNA (Twist Bioscience)

For the clinical SARS-CoV-2 positive samples (*n =* 22), inStrain classification was successful for all samples and all BCMs, except in two cases with no high-quality reads post-filtering and one RVI-seq sequencing failure (Fig. 5a). Interestingly, the RVI-seq method detected a strong inStrain signal in one SARS-CoV-2–negative diagnostic specimen (Fig. 5a). However, this sample exhibited only partial genome coverage (59% at 1 × sequencing depth and 4% at 30 × sequencing depth; Fig. 5c) and was located adjacent to wells containing SARS-CoV-2–positive samples with high viral loads. In addition, sequence type mapping was unsuccessful and did not match neighbouring positive samples. Given the limited and uneven genome coverage, the data do not support reliable assessment of genetic variation. Taken together, these observations suggest that the detected signal is most consistent with well-to-well contamination (carryover), rather than true biological presence of SARS-CoV-2 that was missed by diagnostic PCR.

Control samples (Twist/NIBSC) performed equivalently across BCMs, except for RSV B at the 100-copies, which was only detected by the RVI-seq method (Fig. 5b). Among SARS-CoV-2 positive samples with the lowest Ct (Ct < 16; *n =* 12/22), all BCMs generated full length genomes, except for a single RVI-seq sample that failed sequencing (Fig. 5c). For the six samples with Ct 23–25 all BMCs produced complete or near complete (≥ 70%) genome coverage at 1 × coverage, while RVI-seq and Illumina 3-plex performed slightly better at 30 × coverage (5/6 samples with ≥ 92% coverage), compared to veSEQ (3/6 samples; ≥ 93% coverage), but samples with partial or no coverage at 30 × were not the same for each method (Fig. 5c). Further comparisons of sample Ct or copy number with an extended set of mapping metrics (% genome coverage at ≥ 1x, ≥ 10x, ≥ 30x; absolute read coverage, and mean coverage), showed highly similar results across BCMs up to Ct 23 and variable performance at higher Cts, with no consistent advantage any one BCM (Fig. 5d).

Reference mapping of Twist synthetic RNA or NIBSC purified RNA for SARS-CoV-2, hCoVs, hPIV1 (Human parainfluenza virus 1), and hPIV2 (Human parainfluenza virus 2), both individually and in mixtures, was achieved at comparable levels across all viral BCMs (Fig. 5e). Full-length genomes were successfully reconstructed for Twist synthetic hCoVs, SARS-CoV-2, and hPIV1 (Fig. 5e). Similarly, NIBSC purified RSV B RNA was detected down to 1,000 copies by all methods, and full-length genome mapping was successful at 1 million for all methods (Fig. 5e). Though given the viral copy number for this RSV control sample was estimated prior to nucleic acid extraction, it is therefore not directly comparable on a one-to-one basis with the dilution series of the other quantified TWIST RNA controls.

### Assessment of differences in de novo assembly and reproducibility

**Fig. 6:**
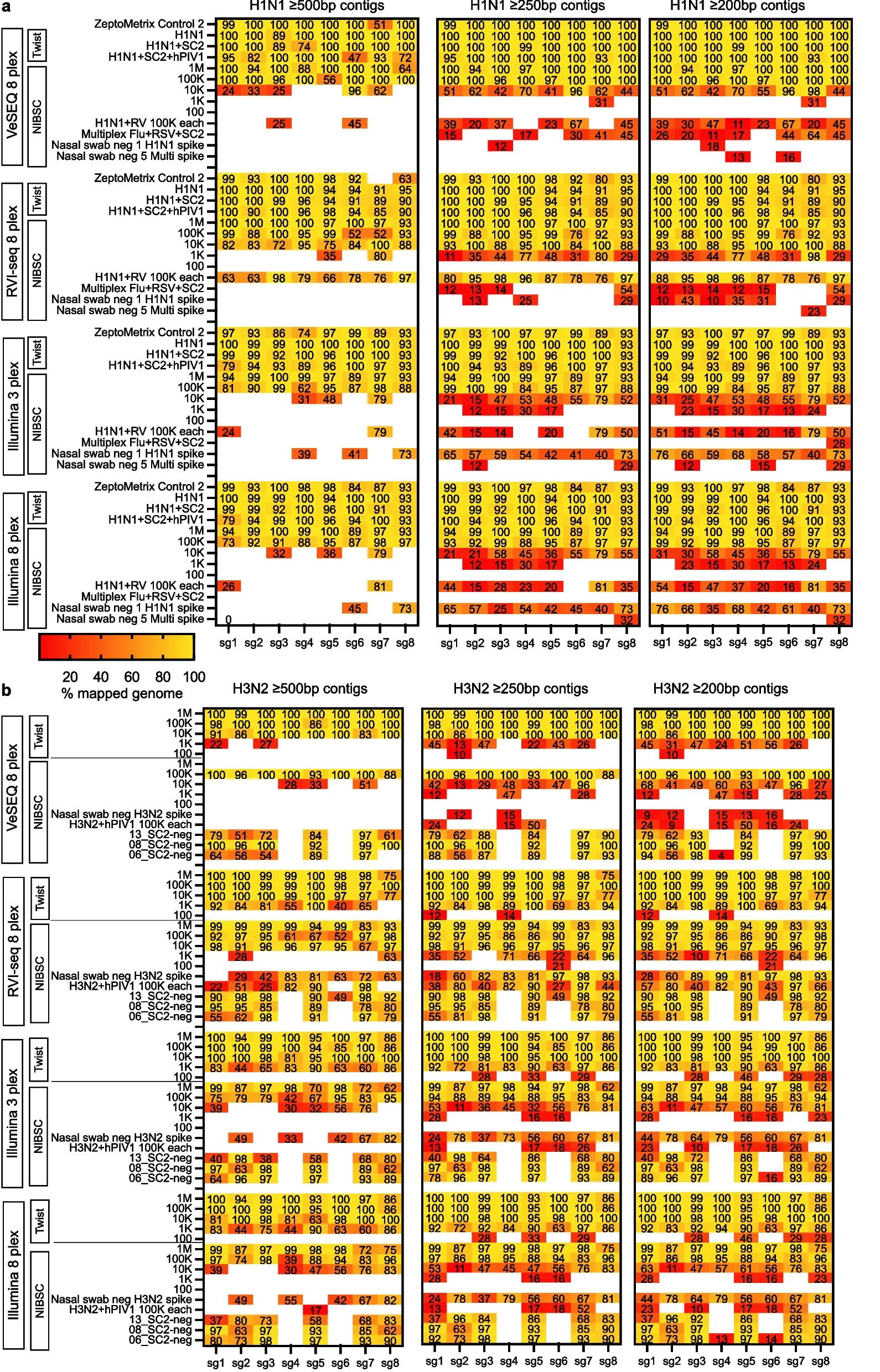
Proportion of H1N1 and H3N2 de novo assembled contigs. The proportion of Influenza A H1N1 (**a**) and H3N2 (**b**) genomic segments 1–8 that were de novo assembled by MetaSPAdes is shown by analysing three different contig lengths: 500 bp, 250 bp, and 200 bp. All eight H1N1 and H3N2 segments (sg1 to sg8) were analysed individually. Abbreviations: SC2 = SARS-CoV2, hPIV1 = human parainfluenza virus 1, RSV = respiratory syncytial virus, RV = rhinovirus. The proportion of mapped genomes is shown in a heatmap, with each cell showing the percentage of the genome mapped for each individual segment

To test for differences in viral BCM performance for de novo assembly we compared data generated for influenza A genomes (H1N1 and H3N2) using three different contig lengths; the MetaSPAdes default minimum length of 500 bp, 250 bp and 200 bp (Fig. 6a-b). The different viral BCMs demonstrated comparable performance for Twist synthetic H1N1 RNA and its mixtures with SARS-CoV-2 and hPIV1, though with some variation between methods observed. No genome coverage was observed in the 100 copy number samples with any of the BCMs. The utilisation of shorter contigs resulted in superior outcomes, particularly for the 1 k copy number samples. Furthermore, three previous clinical diagnosed SARS-CoV-2 negative samples were identified as positive for H3N2 by bait capture enrichment.

We next utilised data from the two independent experiments comparing the RVI-seq (8-plex), veSEQ (8-plex) and Illumina (3-plex) methods to assess reproducibility of results. Focusing on reference mapping for the H3N2 Twist dilution series, all three methods consistently generated full length (98–100%) genome coverage at 30 × for the 1 million, 100 k, and 10 k samples (Additional file 7: Fig. S5), though the pilot 1 Illumina 3-plex run with the high MS2 spike in (noted above) showed some lower coverage for some segments (92–97%). Greater variations were observed for the 1 k and 100 copy samples. Overall, this demonstrated a high degree of reproducibility between the three methods.

### Optimisation of the RVI-seq method for use at scale

Given that there were no major differences between the three viral BCMs evaluated, and established experience translating the RVI-seq method to existing infrastructure for automation, we opted to take the RVI-seq method forward for use at scale. To reduce costs and facilitate automation at scale, we tested the cDNA synthesis steps of the RVI-seq method at 1X reaction volume without reducing sample input volume. Prior tests with the RVI-seq method were scaled to 1.6X to standardise sample input volumes across the three methods; use of a minimised 1X reaction volume would save reagents and thus costs and would facilitate automation using established workflows. As we also observed that the RVI-seq method tended to have a smaller insert size, we increased the first strand synthesis incubation time from 15 to 50 min. Additionally, we assessed the effect of applying a more stringent solid-phase reversible immobilization (SPRI) bead:sample ratio after post-capture PCR. Samples used were a selection of Twist synthetic RNAs including H3N2, SARS-CoV-2 and hCoVs, a purified RSV A (AmpliRun) dilution series, and a selection of SARS-CoV-2 positive clinical samples. We first compared the differences between high-quality (HQ), low-quality (LQ), and human reads for the two different cDNA synthesis reaction volumes (1X and 1.6X), combined with the ‘standard’ or ‘stringent’ SPRI cleanups (Additional file 8: Fig. S6). No significant differences were observed in the relative abundances of HQ paired end reads between standard and stringent SPRI conditions, at either 1X or 1.6X reaction volumes (Kruskal–Wallis with Dunn’s post hoc test *P =* 0.6462).

**Fig. 7:**
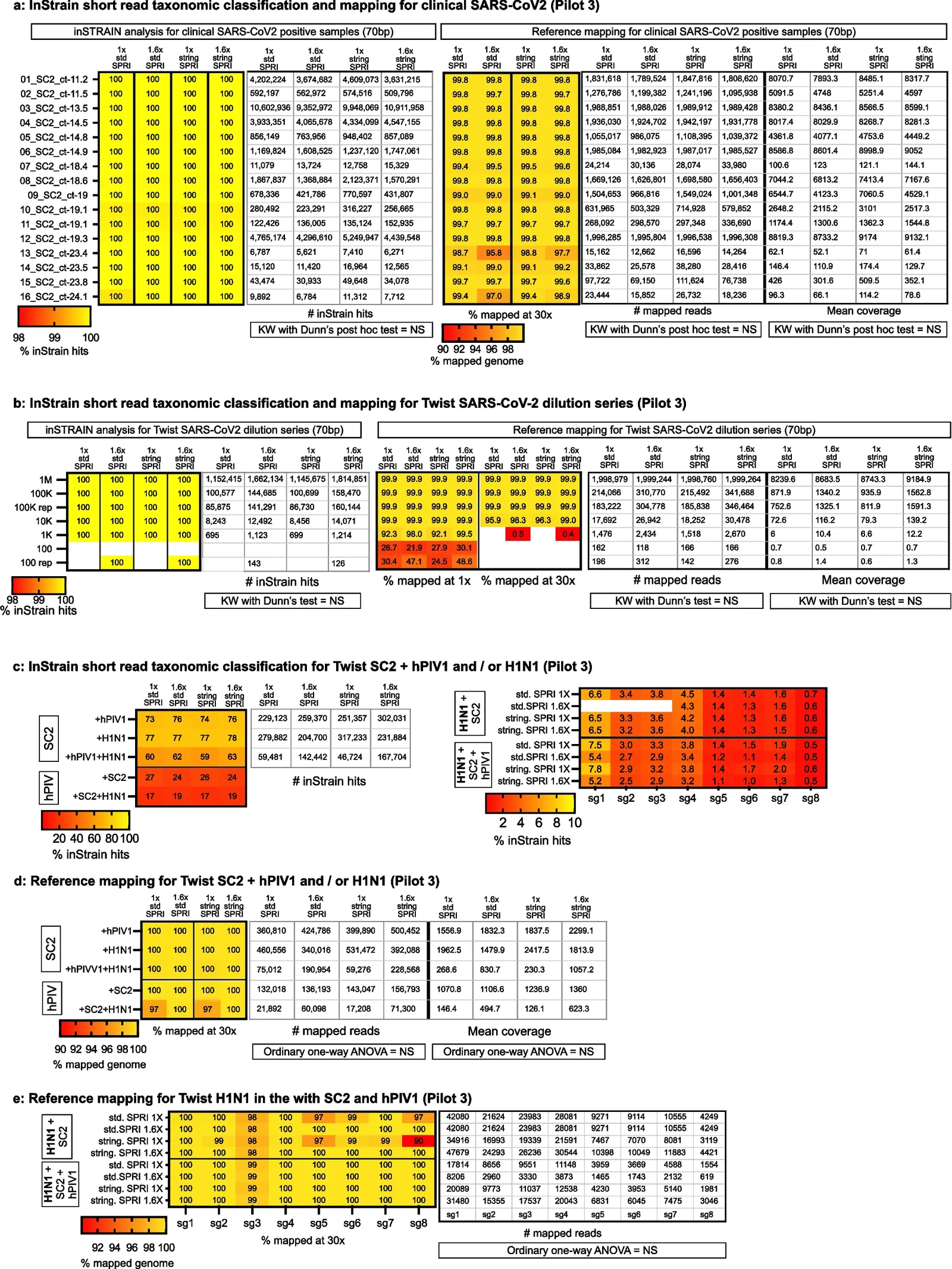
Short-read taxonomic classification and reference mapping of clinical SARS-CoV-2 and Twist synthetic RNA mixtures. High-quality paired-end reads (≥ 70 bp) generated from four laboratory conditions using the RVI-seq bait capture protocol were taxonomically classified using inStrain against the NCBI viral reference database (build 2023–05–09) and mapped to references using Minimap2. Two different SPRI conditions (standard and stringent) were tested in combination with 1X or 1.6X scale cDNA synthesis reaction volumes. **a** Relative inStrain abundances and reference mapping results for 16 SARS-CoV-2 (SC2)-positive clinical samples. The tables show the total number of inStrain hits, the number of mapped reads and the mean coverage. **b** Relative inStrain abundances and reference mapping across a Twist SC2 RNA dilution series. The mapping metrics at 1 × and 30 × sequencing depth are shown in the adjacent tables. **c** Relative inStrain abundances for Twist SC2 samples spiked with hPIV1 and/or influenza A (H1N1). **d** Reference mapping of the same spiked Twist SC2 samples as in **c**. **e** Reference mapping of the eight influenza A H1N1 segments in samples containing SC2 alone or SC2 + hPIV1. The mapped read counts are shown in the adjacent table. In **c** and **e** The H1N1 name in bold in the mixed population corresponds to the value shown in the heat map. Statistical differences in mapped reads and mean coverage across the four methods were assessed using Kruskal–Wallis tests with Dunn’s post hoc test for non-parametric data and one-way ANOVA for parametric comparisons. Mapping metrics were generated using Qualimap v2.3 and heatmaps and statistical analyses were performed using GraphPad Prism 10 (v10.3.1, macOS). Abbreviations: Ct: PCR cycle threshold for SARS-CoV-2 detection, Dilution series: 1 M: one million copies; K: thousand copies; hPIV1: human parainfluenza virus 1; Twist: synthetic RNA (Twist Bioscience)

**Fig. 8:**
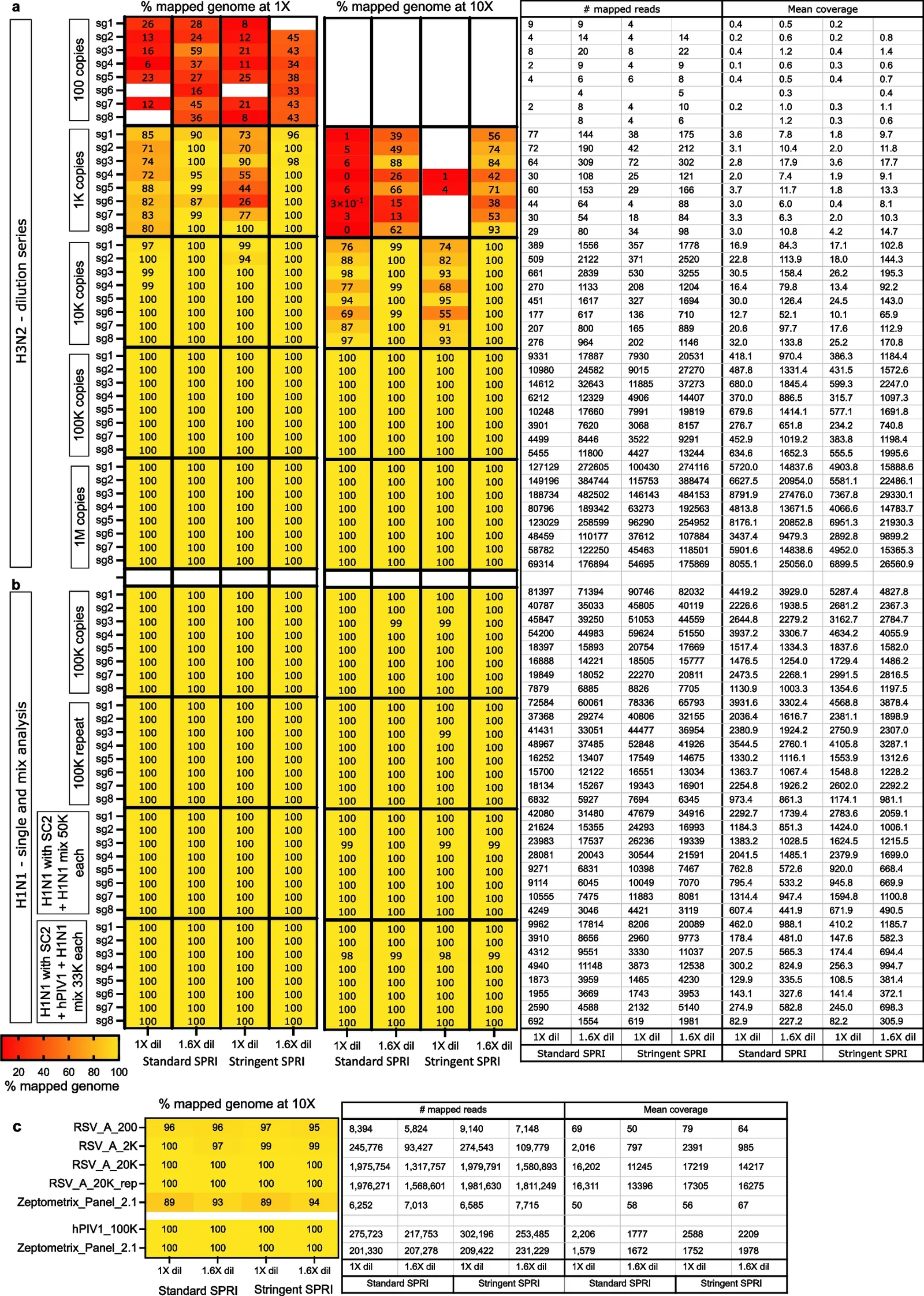
Comparison of reference mapping for the H3N2 dilution series, H1N1 mixed population, RSV-A and hPIV1 by reaction volume and SPRI condition. Two different SPRI conditions (standard and stringent) were tested in combination with 1X or 1.6X scale cDNA synthesis reaction volumes. **a** A heatmap showing the percentage of the genome mapped from the H3N2 dilution series and H1N1 samples at 1X and 10X sequencing depth. The number of mapped reads and mean coverage are presented in the table. The H3N2 dilution series ranged from 1 million to 100 copies. **b** The H1N1 sample was added at a concentration of 100,000 copies, as well as at smaller concentrations in equal proportions, for both the mixture with SC2 (SARS-CoV-2) and the mixture with hPIV1 + SC2. **c** RSV-A was tested at concentrations of 200, 2,000 and 20,000 copies (see Fig. 5c). hPIV1 was tested at a concentration of 100 k copies. Abbreviations: SPRI = solid-phase reversible immobilisation

For the 16 SARS-CoV-2 clinical samples with a range of Ct values (11.2 to 24.1), there were no significant differences in the number of inStrain hits, mapped reads, or mean coverage across the two reaction volumes or SPRI conditions (Fig. 7a). inStrain analysis equivalent across the 4 conditions, and reference-based mapping showed high concordance at 30 × depth (Fig. 7a) and consistent for each dilution down to 1,000 genome copies (Fig. 7b). However, for the 100-copy input, only the 1.6X reaction volume produced a detectable inStrain signal, and mapping at 1 × depth also performed better under this condition (Fig. 7b). In samples containing SARS-CoV-2 mixed with either influenza A (H1N1) or human parainfluenza virus 1 (hPIV1), both viruses were sequenced at high coverage, with generally comparable results across the four conditions. In most cases, all eight H1N1 genome segments were successfully identified by inStrain (Fig. 7c). Similarly, reference-based mapping for these samples was highly consistent, with no statistically significant differences in the number of mapped reads or mean coverage across the four laboratory conditions (Fig. 7d–e). Additional inStrain analysis for human coronavirus, H3N2 dilution series, H1N1, RSV A dilution series, and hPIV1 was highly similar across the four laboratory conditions (Additional file 9: Fig. S7). Additional reference mapping across the four different laboratory conditions for the H3N2 dilution series and for H1N1 alone or mixed with SARS-CoV-2 and/or hPIV1 did not reveal consistent differences between the different conditions (Fig. 8 a-b).

Analysis of the ZeptoMetrix controls (ZeptoMetrix Panel 2.1 in Additional file 9: Fig. S7 and Fig. 8c) containing a mix of respiratory viruses (including hCoVs, adenovirus, influenza A &B, RSV, parainfluenza), likewise yielded comparable results in terms of the total number of high quality reads, and inStrain hits for the range of viral species present across the two reaction volumes and two SPRI conditions.

We further compared various mapping metrics across the four different laboratory conditions for 16 clinical SARS-CoV-2 samples with matching qPCR Ct values (Additional file 10: Fig. S8). These metrics included the percentage of mapped reads at 1 ×, 10 ×, and 30 × depth, the total number of mapped reads, and the mean coverage. The percentage of genome mapped was highly consistent across all four conditions, regardless of Ct value (ranging from 11.2 to 24.1). Only very minor variability was observed in the total number of mapped reads and mean coverage. Overall, both reaction volumes (1X and 1.6X) gave very similar mapping and coverage results across the board, and we found no significant difference between the stringent SPRI and the standard.

**Fig. 9:**
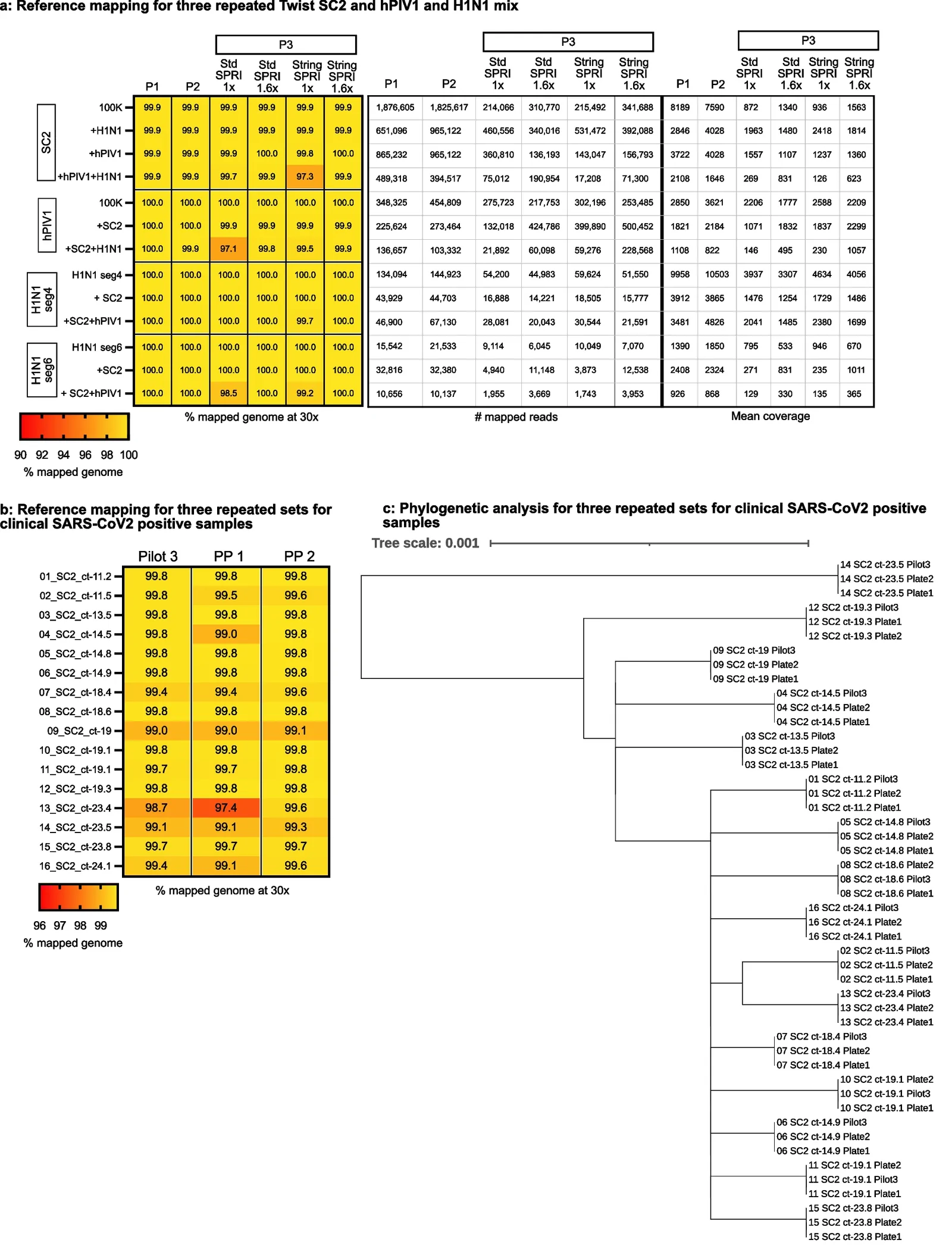
Reproducibility of RVI-seq bait capture across three independent experiments using clinical SARS-CoV-2 samples and synthetic RNA mixtures. High-quality paired-end reads (≥ 70 bp) from matched samples processed in pilot studies 1, 2 and 3 were reference mapped using Minimap2. **a** Reference mapping results at 30 × depth for Twist synthetic SARS-CoV-2 RNA in the absence or presence of hPIV1 and/or influenza A (H1N1) across three independent experiments. The reference mapping results for H1N1 segments 4 (hemagglutinin, HA) and 6 (neuraminidase, NA), both in the absence and presence of SC2 and/or hPIV1, are also shown. Adjacent tables display the number of mapped reads and mean coverage. **b** Reference mapping results at 30 × depth for 16 SARS-CoV-2-positive clinical samples across three replicate experiments. Samples from Pilot 3 were compared to the same samples processed on two additional production plates (PP1 and PP2). **c** A maximum likelihood phylogenetic tree generated with MAFFT confirmed 100% reproducibility in consensus genome sequences across all three experiments for the 16 SARS-CoV-2-positive samples. Samples shown in (a) were obtained from three sources: Pilot 1 (P1), Pilot 2 (P2) and Pilot 3. The latter tested two solid-phase reversible immobilisation (SPRI) bead conditions — standard and stringent — combined with either a 1 × or a 1.6 × scale cDNA synthesis reaction volume. Reference mapping was performed using Minimap2, and mapping metrics were calculated using Qualimap v2.3. Heat maps and statistical visualisations were generated using GraphPad Prism 10 (v10.3.1, macOS). Abbreviations: Ct: PCR cycle threshold for SARS-CoV-2 detection; hPIV1: human parainfluenza virus 1; Twist: synthetic RNA (Twist Bioscience). In pilot experiment three (P3), four laboratory conditions were compared. Two different SPRI conditions (standard and stringent) were tested in combination with either 1X or 1.6X scale cDNA synthesis reaction volumes

We further investigated reproducibility of the RVI-seq data by comparing matched samples from Pilot 3 with data from Pilot 1 and Pilot 2. We compared the reference mapping for Twist synthetic RNA for SARS-CoV-2, hPIV1, and influenza A H1N1 either alone or mixed. The percentage of genome mapped at 30 × sequencing depth was highly consistent across replicates and mixed samples (Fig. 9a). Sixteen clinical SARS-CoV-2 samples that had previously been processed in Pilot 3 were compared with two additional repeats from two other non-pilot plates. This comparison revealed high reproducibility, across the range of viral loads as approximated by qPCR Ct (Fig. 9b and c).

Finally, we analysed additional clinical influenza A & B and RSV A & B samples using reference-based mapping to further validate the RVI-seq BCM pipeline. Twelve clinical H1N1 samples were successfully mapped to the A/California/2009 reference strain (Additional file 11: Fig. S9a), with the exception of two samples that showed a dropout of either segment 3 or 6. Seven H3N2 samples were successfully mapped to the New York/2003 reference strain, although one sample exhibited slightly reduced coverage across all eight segments at 10 × depth (Additional file 11: Fig. S9b). For influenza B, five out of eight samples achieved 90–100% mapping of the different fragments at 30 × depth, while the remaining three samples showed lower mapping success due to a reduced number of available reads (Additional file 11: Fig. S9c).

All synthetic RSV A and RSV B genomes were successfully mapped, with a minimum of 96% coverage down to 200 copies at 10 × depth (Additional file: Fig. S10). Most clinical RSV A samples were mapped with a minimum of 97% genome coverage at 10 × depth. Three samples showed lower mapping performance due to a reduced number of available reads for mapping (ranging from 332 to 3810). Two RSV A samples failed to map entirely because no reads were used for mapping despite high number of HQ R1R2 paired end reads available. Of the 16 clinical RSV B samples, 12 were successfully mapped at 1 × depth with at least 97% genome coverage; at 10 × depth, mapping ranged from 54 to 100% of the genome. Two samples failed mapping at 10 × and 30 × depth because of very low number of reads (n2) used for mapping (Additional file 12: Fig. S10).

Further validation of failed samples using Kraken2 confirmed the initial inStrain results. Across all RSV-negative samples shown in the heatmap, a high proportion of reads were unclassified (ranging from ~ 71% to > 97%), with only a small fraction assigned to viral taxa and no detectable RSV reads. These findings suggest that sequencing failure is most consistent with low abundance of target viral RNA and a high background of non-target material, resulting in insufficient RSV-specific reads for successful bait capture and downstream mapping. Although these samples were positive by diagnostic PCR, this discrepancy likely reflects the higher sensitivity of PCR compared to capture-based sequencing, particularly at low viral loads.

## Discussion

In this study, we evaluated three distinct viral bait capture methods: veSEQ ^23^ Illumina ^22^, and an ‘in-house’ method known as RVI-seq. We found that all three methods had similar levels of performance despite using different library preparations and two different bait panels. They all sequenced a broad range of respiratory viruses successfully, and therefore all have equal potential for genomic surveillance of respiratory viruses, including sequencing multiple different species in the same sample in the case of co-infections.

Across the three pilot experiments and subsequent production runs, several consistent technical patterns emerged. Successful genome recovery was strongly associated with the proportion of target viral reads, with samples containing high levels of non-target or unclassified reads consistently failing to yield complete genomes. This highlights that, despite comparable overall performance between methods, sequencing success is primarily driven by input sample quality rather than the choice of capture approach. Together, these findings informed the selection of the RVI-seq method for larger-scale application, while also defining practical constraints for its use in clinical and surveillance settings.

Multi-pathogen sequencing is a general feature of BCM, which offers an important advantage over single pathogen assays, such as amplicon assays, because it allows study of co-infections and the development of more universal approach desirable for pandemic preparedness and surveillance of endemic diseases. Although we only evaluated baits for viral pathogens, there is no reason that the same methodology couldn’t be adapted to include both viral and bacterial pathogens (e.g. *Streptococcus pneumoniae*). Alternative approaches to bait capture include metagenomic sequencing with depletion of the human component, that has been successfully used in pneumonia patients [53], though a recent study demonstrated the superior sensitivity of targeted bait capture methods over untargeted metagenomics [22].

Using dilution series, we demonstrated that viral detection for a broad range of respiratory viruses including SARS-CoV-2, influenza and RSV was consistently possible as low as 100 copies, and in some cases enabled partial mapping of the whole genome, which demonstrates the high degree of sensitivity of all three methods. We consistently found that full length genomes with 10 × coverage suitable for phylogenetic analysis were generated at ~ 10 k copies by all three method, though it is likely that full-length genomes would be generated below this threshold as thousand copies of synthetic SARS-CoV-2 reproducibly generated > 1,000 reads (Fig. 7b). Ten thousand copies is approximately the viral load in clinical samples with a qPCR Ct of ~ 27 [54, 55], demonstrating that the three bait capture methods show comparable performance to amplicon-based methods [56, 57]. In our analysis of clinical SAR-CoV-2 samples, we were able to generate full-length genomes for all samples we sequenced with a Ct ranging from 11–25 (though it should be noted that we didn’t attempt higher Cts).

Again, the reproducibility of these results across multiple independent experiments demonstrated the robustness of the methods. Similarly bait capture–enriched libraries sequenced with Oxford Nanopore Technologies (ONT) has also reported good sensitivity at high viral loads (60,000 gc/ml) [22], and the veSEQ protocol has been applied to HIV plasma samples, exactly as described here, but with a final step to convert the enriched pooled libraries for PacBio, rather than illumina, achieving median read lengths of 1500 nt per sample.

Despite the current high costs of probe-based hybrid-capture methods, primarily due to the expense of probes, these technologies hold great promise in enabling the simultaneous enrichment of genomic sequences from multiple pathogens, including large viral genomes, such as those of human herpesviruses [58, 59], as well as bacterial genomes like *Mycobacterium tuberculosis* [60]. In terms of the costs per sample, we evaluated the impact that two ways of cost reductions can be achieved. For the Illumina method, we found that pooling at 8-plex rather than the manufacturer recommended 3-plex has no major impact on sensitivity, highlighting further opportunity for cost reductions by increasing the number of samples pooled for bait capture. The veSEQ protocol routinely uses 96-plex capture for HIV and SARS-CoV-2 sequencing producing read counts that are log-linear correlated with sample viral load. We also demonstrate that further optimisations to reduce costs by minimisation of reaction volumes for RVI-seq to reduce reagent usage was possible without reduction in performance. Of note, the veSEQ protocol has also been successfully miniaturised to one-quarter of the manufacturer recommended reaction volume, also using non-stringent SPRI bead concentration of nucleic acid extracts prior to cDNA synthesis (SMARTer Pico Mamamllian v2 Kit) in a non-robotic workflow [20]. A high concentration (4.8 × 10^6^ copies) of spike-in with the MS2 phage in the Illumina method affected the sensitivity of sequencing, which highlights that caution should be applied to concentrations when using spike-in controls. Further systematic optimisations to reduce reagent usage can undoubtedly be achieved providing opportunity for additional cost reductions that will be critical for global use at scale.

The rapid evolution and diversity of human pathogens, coupled with dynamic host–pathogen interactions, present considerable challenges in mitigating the impact of infectious diseases. Traditional short-read classification tools, such as Kraken [39, 40] and inStrain [45], provide useful insights but are limited in their ability to fully capture the extent of pathogen genomes. In contrast, reference-dependent mapping remains an optimal approach for assessing the presence and extent of a given genome [61–63]. However, this method assumes the availability of a reference genome, which may not always be the case for newly emerging pathogens. As an alternative, de novo assembly of short reads offers a more comprehensive approach, enabling detailed genome analysis even without a predefined reference. In our evaluation of bait-captured short reads for de novo assembly, we found that all three methods performed well with some differences. The supplementary results section (Additional File 6) provides additional details on these de novo assembly outcomes.

The emergence of SARS-CoV-2 [64] clearly highlighted the critical role for routine surveillance of respiratory samples in generating new biological insights and public health monitoring. Likewise, the early challenges faced in responding to the Ebola virus epidemic (2013–2016) [65] further emphasize the need for high-throughput technologies capable of providing timely genomic insights. Establishing global genomic surveillance with the capability to spot novel or divergent respiratory viruses is urgently required. Bait capture, although not true metagenomic sequencing, does have the advantage that is tolerant to a certain degree of mismatches in the baits [66] and due to the conserved components of viral genomes (e.g. RNA-dependent RNA polymerase (RdRp) gene in coronaviruses) mean that even if attempting to sequence a divergent or novel virus there is a reasonable likelihood that at least a partial genome sequence will be generated [67, 68]. Indeed, veSEQ recovered near complete SARS-CoV-2 genomes using probes incorporating the SARS-CoV-1 reference genome [21]. Additionally, all BCMs generate metagenomic libraries at an interim step, which can be sequenced directly, without enrichment, in cases where highly divergent pathogens are expected or partially detected in a bait captured sequence.

Bait capture approaches and shotgun metagenomic approaches are less affected by primer mismatch compared to amplicon-based sequencing which may be particularly sensitive to viral genetic drift and shift, as mutations arising in primer binding regions can lead to reduced amplification efficiency or complete amplicon dropout. This phenomenon was widely observed during the COVID-19 pandemic, where successive SARS-CoV-2 variants (e.g., Delta and Omicron) necessitated repeated redesigns of ARTIC primer schemes to maintain genome coverage and sequencing accuracy [69, 70].

Overall, we provide a comprehensive benchmarking of three different viral bait capture methods which had similar and consistent performance demonstrating the utility of all three methods, providing users with confidence that any of the methods will yield useful data.

Although challenges remain, particularly in terms of cost, we demonstrate cost reductions can be made without affecting performance. In conclusion, we show in general that bait capture for respiratory viruses has great potential for routine global surveillance, offering the ability to rapidly characterize multiple known and emerging viruses with the same assay.

The longer turnaround time for bait capture analysis (approximately 5–7 days), compared to significantly shorter PCR-based diagnostic tests or other more rapid sequencing methos, currently limits its use to secondary applications such as viral surveillance and genomic characterization, though ongoing improvements in turnaround times mean genomics is being increasing used as part of diagnostic pathways [71, 72].

In our study, 10,000 copies (approximately Ct ~ 27 in our calibration) consistently produced high genome recovery across methods. We agree that performance declines at very low input levels (≤ 1,000 copies; approximately Ct ~ 30), as shown in Fig. 6b.

It is important to distinguish between diagnostic RT-PCR sensitivity and sequencing performance. Diagnostic assays may generate detectable amplification signals at Ct values of 40–45; however, detection at these late cycles does not necessarily translate into sufficient template quantity for robust whole-genome sequencing. Amplicon-based sequencing methods also typically experience reduced genome completeness and increased dropout at high Ct values Ct > 30. Additionally, we recognise that inclusion of clinical samples with higher Ct values would have further strengthen this comparison.

## Conclusion

Our study demonstrates that viral bait capture sequencing is a robust and highly reproducible platform for comprehensive respiratory virus genomics. By showing equivalent performance across three independent workflows and identifying practical strategies to reduce assay costs, we provide an evidence-based framework for large-scale implementation of bait capture sequencing in routine genomic surveillance. As respiratory pathogens continue to evolve and novel viruses emerge, scalable multi-pathogen sequencing approaches such as these will be critical for strengthening global infectious disease surveillance and improving preparedness for future epidemics and pandemics.

## Supplementary Information

Additional file 1: Table S1. Sample Type, Sample Name, Sequence file ID for pilot studies 1, 2 and 3 and for production plates 1 and 2.

Additional file 2: Fig. S1. Number of raw reads across the two replicated sequencing runs were very similar in the Pilot 1 experiment.

Additional file 3: Fig S2. Higher classification differences detected between the three different bait capture methods in pilot 1 study.

Additional file 4: Fig S3. Pilot 1 - Viral Bait Capture inStrain analysis demonstrating the high abundance of spiked-in phage MS2 in the Illumina dataset.

Additional file 5: Fig S4. Insert size distribution is smallest in the RVI-seq bait capture method.

Additional file 6. Supplementary Results. Supplementary Results for de-novo assembly and inStrain optimisation.

Additional file 7: Fig S5. Reference mapping for H3N2 Twist dilution series demonstrates consistent reproducibility.

Additional file 8: Fig S6. Relative frequencies of HQ paired end, unmatched reads, human reads and low-quality reads across the four different SPRI methods (Pilot 3).

Additional file 9: Fig S7. inStrain classification for hCoV, Flu H3N2 and H1N1, HPIV1, RSV A and ZeptoMetrix control samples.

Additional file 10: Fig S8. Correlation between Ct value and reference mapping performance metrics.

Additional file 11: Fig S9. Reference mapping for clinical Influenza A and B viruses.

Additional file 12: Fig S10. Validation of synthetic and clinical RSV A and B samples.

## Data Availability

The raw sequence data used in pilot experiment 1–3 and production plates 1–2 have been deposited in the European Nucleotide Archive (ENA) under study accession number ERP191543. [73] 74. Wellcome Sanger Institute. European Nucleotide Archive (ENA) https://www.ebi.ac.uk/ena/browser/submit. ENA; 2026. https://www.ebi.ac.uk/ena/browser/view/ERP191543.

## Abbreviations

BCM/BCMs: Bait Capture Method(s) (also written as viral BCMs)
cDNA: Complementary DNA
DNA: Deoxyribonucleic Acid
dsDNA: Double-stranded DNA
gDNA: Genomic DNA
MS2: Enterobacteria phage MS2 (used as spike-in control)
NCBI: National Center for Biotechnology Information
NIBSC: National Institute for Biological Standards and Control (UK)
PCR: Polymerase Chain Reaction
PLEX: Multiplex (referring to sample multiplexing level)
RNA: Ribonucleic Acid
Ct: PCR cycle threshold value
RVI-seq: Respiratory Virus Identification Sequencing (bait capture method)
SARS-CoV-2 (SC2): Severe Acute Respiratory Syndrome Coronavirus 2
H1N1 H3N2: Influenza virus A subtype
RSV A: Respiratory synthetical virus
hPIV: Human Parainfluenza Virus
hCoV: Human Corona Virus
PP1 and PP2: Production plate 1 and 2

## References

[1] Miller RR, Montoya V, Gardy JL, Patrick DM, Tang P. Metagenomics for pathogen detection in public health. Genome Med. 2013;5:81. 10.1186/gm485. PMC3978900 24050114

[2] Gu W, Miller S, Chiu CY. Clinical metagenomic next-generation sequencing for pathogen detection. Annu Rev Pathol Mech Dis. 2019;14:319–38. 10.1146/annurev-pathmechdis-012418-012751. PMC6345613 30355154

[3] Jacob JJ, Veeraraghavan B, Vasudevan K. Metagenomic next-generation sequencing in clinical microbiology. Indian J Med Microbiol. 2019;37:133–40. 10.4103/ijmm.IJMM_19_401. 31745012

[4] Deurenberg RH, Bathoorn E, Chlebowicz MA, Couto N, Ferdous M, García-Cobos S, et al. Application of next generation sequencing in clinical microbiology and infection prevention. J Biotechnol. 2017;243:16–24. 10.1016/j.jbiotec.2016.12.022. 28042011

[5] Mate SE, Kugelman JR, Nyenswah TG, Ladner JT, Wiley MR, Cordier-Lassalle T, et al. Molecular evidence of sexual transmission of Ebola virus. N Engl J Med. 2015;373:2448–54. 10.1056/NEJMoa1509773. PMC4711355 26465384

[6] Kugelman JR, Wiley MR, Mate S, Ladner JT, Beitzel B, Fakoli L, et al. Monitoring of Ebola virus Makona evolution through establishment of advanced genomic capability in Liberia. Emerg Infect Dis. 2015;21:1135–43. 10.3201/eid2107.150522. PMC4816332 26079255

[7] Quick J, Loman NJ, Duraffour S, Simpson JT, Severi E, Cowley L, et al. Real-time, portable genome sequencing for Ebola surveillance. Nature. 2016;530:228–32. 10.1038/nature16996. PMC4817224 26840485

[8] Grubaugh ND, Ladner JT, Kraemer MUG, Dudas G, Tan AL, Gangavarapu K, et al. Genomic epidemiology reveals multiple introductions of Zika virus into the United States. Nature. 2017;546:401–5. 10.1038/nature22400. PMC5536180 28538723

[9] Faria NR, Azevedo RdoSdaS, Kraemer MUG, Souza R, Cunha MS, Hill SC, et al. Zika virus in the Americas: early epidemiological and genetic findings. Science. 2016;352:345–9. 10.1126/science.aaf5036. PMC4918795 27013429

[10] Sardi SI, Somasekar S, Naccache SN, Bandeira AC, Tauro LB, Campos GS, et al. Coinfections of Zika and Chikungunya Viruses in Bahia, Brazil, Identified by Metagenomic Next-Generation Sequencing. J Clin Microbiol. 2016;54:2348–53. 10.1128/JCM.00877-16. PMC5005514 27413190

[11] Tang W, Kim J, Lee RT, Maurer-Stroh S, Renia L, Tay MZ. SARS-CoV-2: lessons in virus mutation prediction and pandemic preparedness. Curr Opin Immunol. 2025;95:102560. 10.1016/j.coi.2025.102560. 40378522

[12] Tegally H, San JE, Cotten M, Moir M, Tegomoh B, Mboowa G, et al. The evolving SARS-CoV-2 epidemic in Africa: Insights from rapidly expanding genomic surveillance. Science. 2022;378. 10.1126/science.abq5358. PMC9529057 36108049

[13] Nasir JA, Kozak RA, Aftanas P, Raphenya AR, Smith KM, Maguire F, et al. A comparison of whole genome sequencing of SARS-CoV-2 using amplicon-based sequencing, random hexamers, and bait capture. Viruses. 2020;12:895. 10.3390/v12080895. PMC7472420 32824272

[14] Mak TM, Stegger M, Mølbak K, Maurer-Stroh S. Pathogen genomics for surveillance and outbreak investigations. In: Fisher D, editor. Infect Dis Emergencies Prep Response. 1st ed. NUS Press; 2025. p. 270-88. 10.56159/emergencies-21. Cited 2025 July 1.

[15] Aggarwal D, Warne B, Jahun AS, Hamilton WL, Fieldman T, Du Plessis L, et al. Genomic epidemiology of SARS-CoV-2 in a UK university identifies dynamics of transmission. Nat Commun. 2022;13:751. 10.1038/s41467-021-27942-w. PMC8826310 35136068

[16] Rose R, Constantinides B, Tapinos A, Robertson DL, Prosperi M. Challenges in the analysis of viral metagenomes. Virus Evol. 2016;2:vew022. 10.1093/ve/vew022. PMC5822887 29492275

[17] Mertes F, ElSharawy A, Sauer S, Van Helvoort JMLM, Van Der Zaag PJ, Franke A, et al. Targeted enrichment of genomic DNA regions for next-generation sequencing. Brief Funct Genomics. 2011;10:374–86. 10.1093/bfgp/elr033. PMC3245553 22121152

[18] Soria-Villalba A, Pesantes N, Jiménez-Hernández N, Pons J, Moya A, Pérez-Brocal V. Comparison of experimental methodologies based on bulk-metagenome and virus-like particle enrichment: pros and cons for representativeness and reproducibility in the study of the fecal human virome. Microorganisms. 2024;12:162. 10.3390/microorganisms12010162. PMC10820677 38257988

[19] Gaudin M, Desnues C. Hybrid capture-based next generation sequencing and its application to human infectious diseases. Front Microbiol. 2018;9. 10.3389/fmicb.2018.02924. PMC6277869 30542340

[20] Bonsall D, Golubchik T, Cesare M de, Limbada M, Kosloff B, MacIntyre-Cockett G, et al. A Comprehensive Genomics Solution for HIV Surveillance and Clinical Monitoring in Low-Income Settings. J Clin Microbiol. 2020;58. 10.1128/JCM.00382-20. PMC7512176 32669382

[21] Lythgoe KA, Hall M, Ferretti L, Cesare M de, MacIntyre-Cockett G, Trebes A, et al. SARS-CoV-2 within-host diversity and transmission. Science. 2021;372. 10.1126/science.abg0821. PMC8128293 33688063

[22] Buddle S, Forrest L, Akinsuyi N, Martin Bernal LM, Brooks T, Venturini C, et al. Evaluating metagenomics and targeted approaches for diagnosis and surveillance of viruses. Genome Med. 2024;16:111. 10.1186/s13073-024-01380-x. PMC11382446 39252069

[23] Montaguth OET, Buddle S, Forrest L, Brooks T, Tutill H, Simmonds P, et al. Nanopore-based hybridization capture approaches for targeted viral metagenomics and whole-genome sequencing. Infect Dis (except HIV/AIDS). 2025. 10.1101/2025.04.08.25325453. Cited 2025 Sept 18.

[24] Mourik K, Sidorov I, Carbo EC, Van Der Meer D, Boot A, Kroes ACM, et al. Comparison of the performance of two targeted metagenomic virus capture probe-based methods using reference control materials and clinical samples. J Clin Microbiol. 2024;62:e00345-24. 10.1128/jcm.00345-24. St. George K, editor. PMC11237577 38757981

[25] NIBSC. The National Institute for Biological Standards and Control. https://nibsc.org. Accessed 22 July 2026.

[26] ZeptoMetrix Panel 2.1. https://www.zeptometrix.com/au/en/nattrol-respiratory-verification-panel-21-24-x-06ml-ea-3073. Accessed 22 July 2026.

[27] TWIST. https://www.twistbioscience.com/products/ngs/synthetic-viral-controls. Accessed 22 July 2026.

[28] Ampheus. https://www.protocols.io/view/probe-based-target-enrichment-of-sars-cov-2-ewov185o7gr2/v1. Accessed 22 July 2026.

[29] protocols.io. https://www.protocols.io/view/rvi-seq-multi-respiratory-virus-sequencing-protoco-e6nvw1b62lmk/v1. Accessed 22 July 2026.

[30] TWIST Bioscience Resources. https://www.twistbioscience.com/resources. Accessed 22 July 2026.

[31] TWIST Viral Enrichment Paneal. https://www.twistbioscience.com/resources/product-sheet/respiratory-virus-research-panel-product-sheet. Accessed 22 July 2026.

[32] Illumina Respiratory Pathogen Panel. https://emea.illumina.com/content/dam/illumina/gcs/assembled-assets/marketing-literature/respiratory-pathogen-panel-table-m-gl-00115/respiratory-pathogen-id-amr-panel-table-m-gl-00115.pdf. Accessed 22 July 2026.

[33] Illumina. Illumina Respiratory Virus Enrichment Kit. https://sapac.illumina.com/products/by-type/sequencing-kits/library-prep-kits/respiratory-virus-oligo-panel.html. Accessed 22 July 2026.

[34] Illumina RNA Prep with Enrichment. https://www.illumina.com/content/dam/illumina/gcs/assembled-assets/marketing-literature/illumina-rna-enrichment-coronavirus-app-note-470-2020-005/illumina-rna-enrichment-coronavirus-app-note-470-2020-005.pdf. Accessed 22 July 2026.

[35] RVI-viral metagenomics pipeline. https://github.com/sanger-pathogens/rvi-viral-metagenomics-pipeline. Accessed 22 July 2026.

[36] Sanger Institute. RVI-viral lens. https://github.com/sanger-pathogens/rvi-viral-lens. Accessed 22 July 2026.

[37] Huttenhower group. Kneaddata. https://github.com/biobakery/kneaddata. Accessed 22 July 2026.

[38] NCBI. Human Reference Genome GRCh38. http://hgdownload.cse.ucsc.edu/goldenPath/hg38/bigZips/. Accessed 22 July 2026.

[39] Lu J, Rincon N, Wood DE, Breitwieser FP, Pockrandt C, Langmead B, et al. Metagenome analysis using the Kraken software suite. Nat Protoc. 2022;17:2815–39. 10.1038/s41596-022-00738-y. PMC9725748 36171387

[40] Wood DE, Salzberg SL. Kraken: ultrafast metagenomic sequence classification using exact alignments. Genome Biol. 2014;15:R46. 10.1186/gb-2014-15-3-r46. PMC4053813 24580807

[41] Lu J, Breitwieser FP, Thielen P, Salzberg SL. Bracken: estimating species abundance in metagenomics data. PeerJ Comput Sci. 2017;3:e104. 10.7717/peerj-cs.104. PMC12016282 40271438

[42] ccb.jhu.edu. Bracken. https://ccb.jhu.edu/software/bracken/index.shtml?t=manual#step1.

[43] PlusPFP database for Kraken. https://genome-idx.s3.amazonaws.com/kraken/k2_pluspfp_20230314.tar.gz. Accessed 22 July 2026.

[44] Ben Langmead. Kraken 2 Ben Langmead’s index site. https://benlangmead.github.io/aws-indexes/k2. Accessed 22 July 2026.

[45] Olm MR, Crits-Christoph A, Bouma-Gregson K, Firek BA, Morowitz MJ, Banfield JF. inStrain profiles population microdiversity from metagenomic data and sensitively detects shared microbial strains. Nat Biotechnol. 2021;39:727–36. 10.1038/s41587-020-00797-0. PMC9223867 33462508

[46] Li H. New strategies to improve minimap2 alignment accuracy. Bioinformatics. 2021;37:4572–4. 10.1093/bioinformatics/btab705. PMC8652018 34623391

[47] Li H. Minimap2: pairwise alignment for nucleotide sequences. Bioinformatics. 2018;34:3094–100. 10.1093/bioinformatics/bty191. PMC6137996 29750242

[48] Danecek P, Bonfield JK, Liddle J, Marshall J, Ohan V, Pollard MO, et al. Twelve years of SAMtools and BCFtools. Gigascience. 2021;10:giab008. 10.1093/gigascience/giab008. PMC7931819 33590861

[49] García-Alcalde F, Okonechnikov K, Carbonell J, Cruz LM, Götz S, Tarazona S, et al. Qualimap: evaluating next-generation sequencing alignment data. Bioinformatics. 2012;28:2678–9. 10.1093/bioinformatics/bts503. 22914218

[50] Nurk S, Meleshko D, Korobeynikov A, Pevzner PA. metaSPAdes: a new versatile metagenomic assembler. Genome Res. 2017;27:824–34. 10.1101/gr.213959.116. PMC5411777 28298430

[51] Bankevich A, Nurk S, Antipov D, Gurevich AA, Dvorkin M, Kulikov AS, et al. Spades: a new genome assembly algorithm and its applications to single-cell sequencing. J Comput Biol. 2012;19:455–77. 10.1089/cmb.2012.0021. PMC3342519 22506599

[52] Mikheenko A, Saveliev V, Gurevich A. MetaQUAST: evaluation of metagenome assemblies. Bioinformatics. 2016;32:1088–90. 10.1093/bioinformatics/btv697. 26614127

[53] Charalampous T, Alcolea-Medina A, Snell LB, Alder C, Tan M, Williams TGS, et al. Routine metagenomics service for ICU patients with respiratory infection. Am J Respir Crit Care Med. 2024;209:164–74. 10.1164/rccm.202305-0901OC. PMC10806431 37938162

[54] Gentilini F, Turba ME, Taddei F, Gritti T, Fantini M, Dirani G, et al. Modelling RT-qPCR cycle-threshold using digital PCR data for implementing SARS-CoV-2 viral load studies. PLoS One. 2021;16:e0260884. 10.1371/journal.pone.0260884. PMC8687578 34928966

[55] Juanola-Falgarona M, Peñarrubia L, Jiménez-Guzmán S, Porco R, Congost-Teixidor C, Varo-Velázquez M, et al. Ct values as a diagnostic tool for monitoring SARS-CoV-2 viral load using the QIAstat-Dx® Respiratory SARS-CoV-2 Panel. Int J Infect Dis. 2022;122:930–5. 10.1016/j.ijid.2022.07.022. PMC9273520 35840097

[56] Kubik S, Marques AC, Xing X, Silvery J, Bertelli C, Maio FD, et al. Recommendations for accurate genotyping of SARS-CoV-2 using amplicon-based sequencing of clinical samples. Clin Microbiol Infect. 2021;27:1036.e1-1036.e8. 10.1016/j.cmi.2021.03.029. PMC8016543 33813118

[57] Charre C, Ginevra C, Sabatier M, Regue H, Destras G, Brun S, et al. Evaluation of NGS-based approaches for SARS-CoV-2 whole genome characterisation. Virus Evol. 2020;6. 10.1093/ve/veaa075. PMC7665770 33318859

[58] McGeoch DJ, Dalrymple MA, Davison AJ, Dolan A, Frame MC, McNab D, et al. The complete DNA sequence of the long unique region in the genome of herpes simplex virus type 1. J Gen Virol. 1988;69:1531–74. 10.1099/0022-1317-69-7-1531. 2839594

[59] Depledge DP, Palser AL, Watson SJ, Lai IY-C, Gray ER, Grant P, et al. Specific capture and whole-genome sequencing of viruses from clinical samples. PLoS One. 2011;6:e27805. 10.1371/journal.pone.0027805. PMC3220689 22125625

[60] Brown AC, Bryant JM, Einer-Jensen K, Holdstock J, Houniet DT, Chan JZM, et al. Rapid whole-genome sequencing of *Mycobacterium tuberculosis* isolates directly from clinical samples. J Clin Microbiol. 2015;53:2230–7. 10.1128/JCM.00486-15. PMC4473240 25972414

[61] Wylezich C, Calvelage S, Schlottau K, Ziegler U, Pohlmann A, Höper D, et al. Next-generation diagnostics: virus capture facilitates a sensitive viral diagnosis for epizootic and zoonotic pathogens including SARS-CoV-2. Microbiome. 2021;9:51. 10.1186/s40168-020-00973-z. PMC7896545 33610182

[62] Maabar M, Davison AJ, Vučak M, Thorburn F, Murcia PR, Gunson R, et al. DisCVR: Rapid viral diagnosis from high-throughput sequencing data. Virus Evol. 2019;5:vez033. 10.1093/ve/vez033. PMC6735924 31528358

[63] Rahimian M, Panahi B. Metagenome sequence data mining for viral interaction studies: Review on progress and prospects. Virus Res. 2024;349:199450. 10.1016/j.virusres.2024.199450. PMC11388672 39151562

[64] Pagani I, Ghezzi S, Alberti S, Poli G, Vicenzi E. Origin and evolution of SARS-CoV-2. Eur Phys J Plus. 2023;138:157. 10.1140/epjp/s13360-023-03719-6. PMC9933829 36811098

[65] Ladner JT, Sahl JW. Towards a post-pandemic future for global pathogen genome sequencing. PLoS Biol. 2023;21:e3002225. 10.1371/journal.pbio.3002225. PMC10393143 37527248

[66] Brown JR, Roy S, Ruis C, Yara Romero E, Shah D, Williams R, et al. Norovirus Whole-Genome Sequencing by SureSelect Target Enrichment: a Robust and Sensitive Method. J Clin Microbiol. 2016;54:2530–7. 10.1128/JCM.01052-16. Tang Y-W, editor. PMC5035417 27487952

[67] Bonsall D, Ansari MA, Ip C, Trebes A, Brown A, Klenerman P, et al. Ve-SEQ: robust, unbiased enrichment for streamlined detection and whole-genome sequencing of HCV and other highly diverse pathogens. F1000Res. 2015;4:1062. 10.12688/f1000research.7111.1. PMC4821293 27092241

[68] Kuchinski KS, Loos KD, Suchan DM, Russell JN, Sies AN, Kumakamba C, et al. Targeted genomic sequencing with probe capture for discovery and surveillance of coronaviruses in bats. Elife. 2022;11:e79777. 10.7554/eLife.79777. PMC9643004 36346652

[69] Ulhuq FR, Barge M, Falconer K, Wild J, Fernandes G, Gallagher A, et al. Analysis of the ARTIC V4 and V4.1 SARS-CoV-2 primers and their impact on the detection of Omicron BA.1 and BA.2 lineage-defining mutations. Microb Genomics. 2023;9. 10.1099/mgen.0.000991. Cited 19 Mar 2026. PMC10210939 37083576

[70] Hunt M, Hinrichs AS, Anderson D, Karim L, Dearlove BL, Knaggs J, et al. Addressing pandemic-wide systematic errors in the SARS-CoV-2 phylogeny. Nat Methods. 2026;23:653–62. 10.1038/s41592-025-02947-1. PMC12982125 41663577

[71] Torres Montaguth OE, Buddle S, Morfopoulou S, Breuer J. Clinical metagenomics for diagnosis and surveillance of viral pathogens. Nat Rev Microbiol. 2026;24:61–75. 10.1038/s41579-025-01223-5. 40804538

[72] Alcolea-Medina A, Snell LB, Humayun G, Al-Yaakoubi N, Ward D, Alder C, et al. Rapid pan-microbial metagenomics for pathogen detection and personalised therapy in the intensive care unit: a single-centre prospective observational study. Lancet Microbe. 2025;6:101174. 10.1016/j.lanmic.2025.101174. 41045941

[73] Wellcome Sanger Institute. European Nucleotide Archive (ENA). https://www.ebi.ac.uk/ena/browser/submit. ENA; 2026. https://www.ebi.ac.uk/ena/browser/view/ERP191543.

