# Supplementary material for: Benchmarking and optimisation of bait-capture metagenomics for sequencing of respiratory viruses at scale
Source: Genome Med. 2026 Jul 30;18:166. doi: 10.1186/s13073-026-01725-8 (PMC13715170; doi:10.1186/s13073-026-01725-8)
Supplement: Supplementary file 1 [file 13073_2026_1725_MOESM10_ESM.pdf]

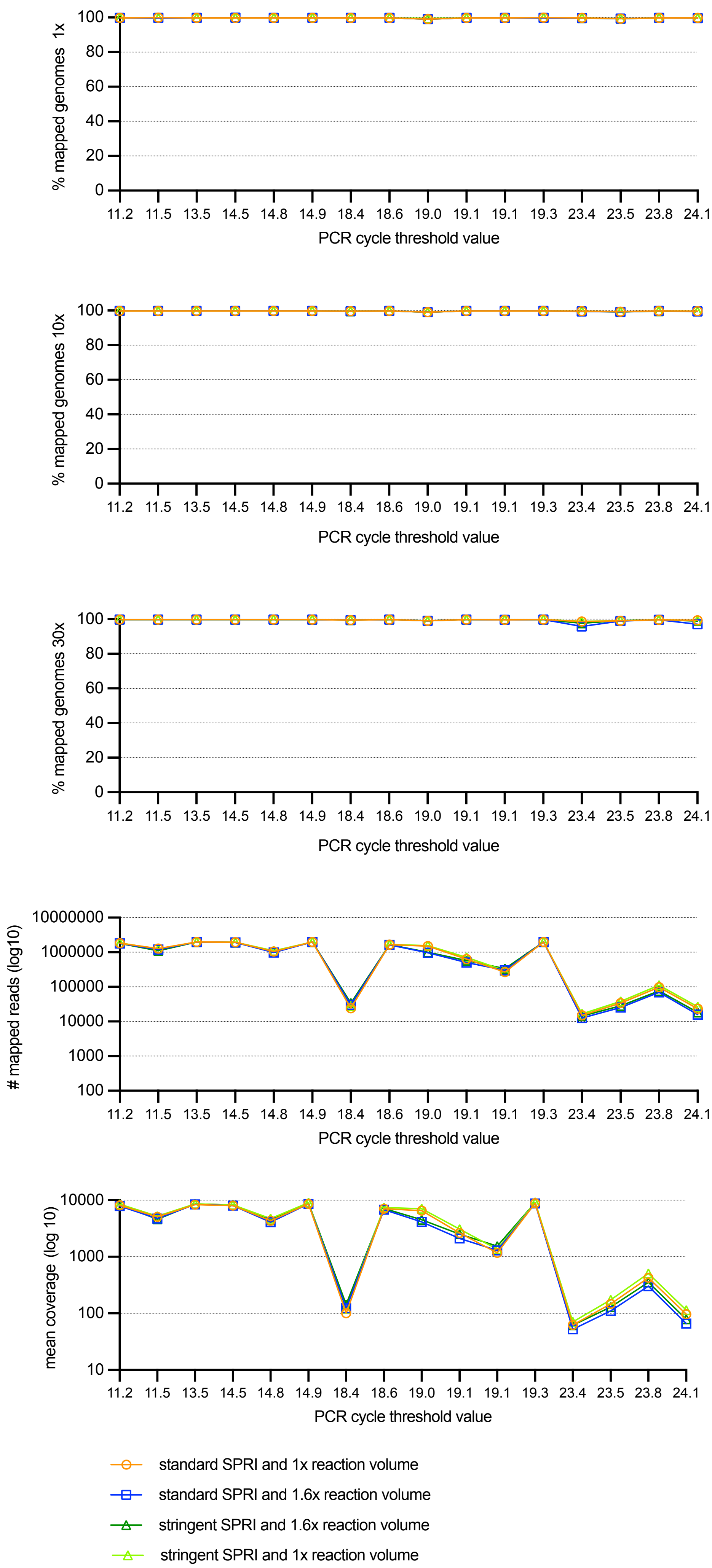

Fig. S8: Correlation between Ct value and reference mapping performance metrics

Relationship between PCR cycle threshold (Ct) values and sequencing outcomes for 16 SARS-CoV-2–positive clinical samples from the Pilot 3 study, processed with the in-house RVI-seq bait capture method. Pilot 3 evaluated two solid-phase reversible immobilisation (SPRI) bead conditions, standard SPRI and stringent SPRI in combination with two cDNA synthesis reaction volumes (1× and 1.6×). Sequencing performance metrics include the proportion of the genome covered at 1×, 10×, and 30× depth, total mapped reads, and mean coverage.
