# Supplementary material for: Benchmarking and optimisation of bait-capture metagenomics for sequencing of respiratory viruses at scale
Source: Genome Med. 2026 Jul 30;18:166. doi: 10.1186/s13073-026-01725-8 (PMC13715170; doi:10.1186/s13073-026-01725-8)
Supplement: Supplementary file 2 [file 13073_2026_1725_MOESM11_ESM.pdf]

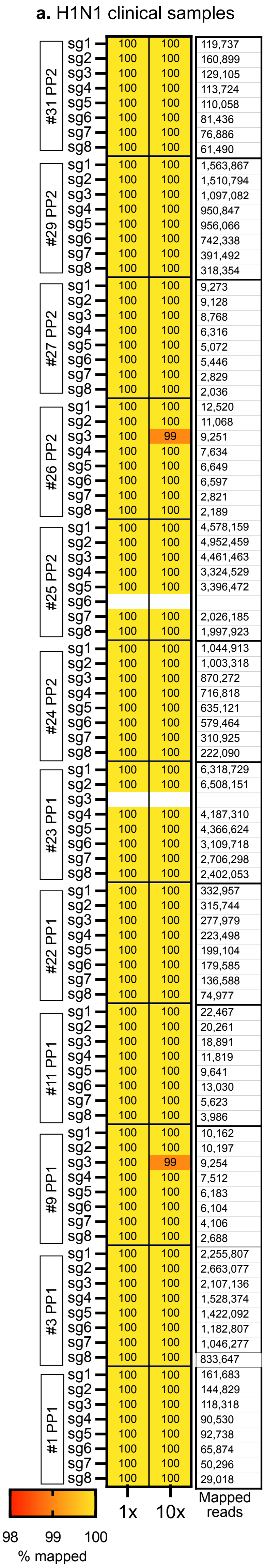

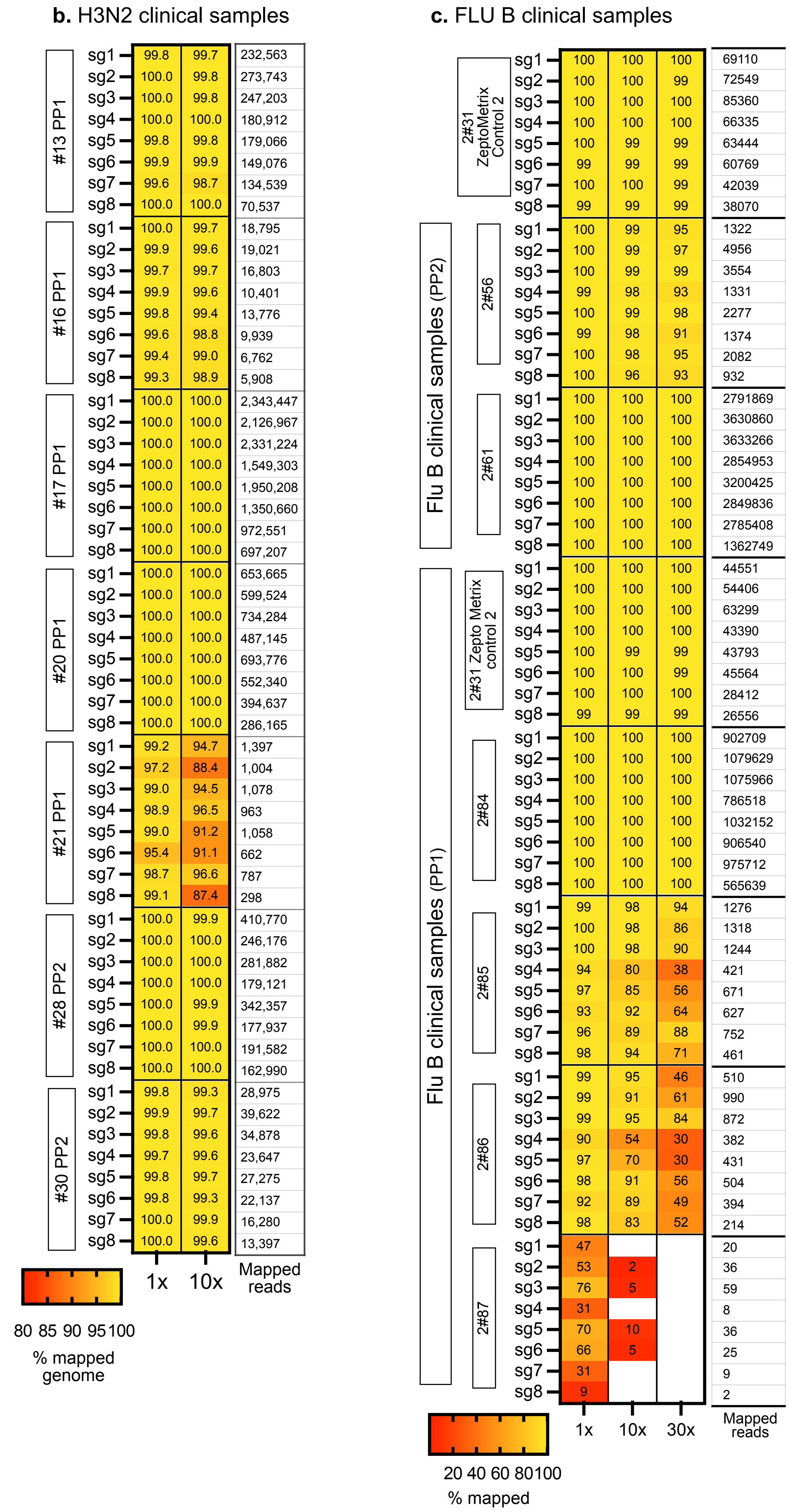

Fig. S9: Reference mapping for clinical Influenza A and B viruses

High-quality paired-end reads with a minimum read length of 70 bp from clinical samples were mapped against reference genomes: (**a**) H1N1 A/California/04/2009, (**b**) H3N2 A/New York/26/2003, and (**c**) B/Brisbane/2008. The proportions of genome coverage across the eight influenza segments at 1× and 10× depth, along with the number of mapped reads, are presented in adjacent tables. Mapping was performed using minimap2, and the proportion of mapped genomes across the segments, as well as the number of mapped reads, were assessed with Qualimap and are visualised in heat maps. Abbreviations:** PP indicates the production plate number. PP1 corresponds to run ID 48367, and PP2 corresponds to run ID 48411.
