# Supplementary material for: Benchmarking and optimisation of bait-capture metagenomics for sequencing of respiratory viruses at scale
Source: Genome Med. 2026 Jul 30;18:166. doi: 10.1186/s13073-026-01725-8 (PMC13715170; doi:10.1186/s13073-026-01725-8)
Supplement: Supplementary file 3 [file 13073_2026_1725_MOESM12_ESM.pdf]

Sample group	Subgroup	Plate	1x	10x	30x	# HQ R1R2 paired reads	# mapped reads	Mean coverage
Synthetic RSV A RNA	20K	P1	100	100	100	5,244,234	5,222,198	49571.36
		P2	100	100	100	1,402,042	1,393,150	13082.01
		P3	100	100	100	3,528,724	3,482,507	32347.1
	20K rep	P1	100	100	100	7,210,646	7,180,700	68111.87
		P2	100	100	100	8,235,376	15,277,267	143533.87
		P3	100	100	100	6,426,622	6,345,318	59226.3
	2K	P1	100	100	100	587,060	585,027	5554.56
		P2	100	99	94	101,046	100,661	946.54
		P3	100	99	98	451,736	446,451	4139.4
	200	P1	100	99	97	18,160	18,007	170.61
		P2	100	96	86	5,018	4,923	46.24
		P3	100	99	97	22,916	22,440	207.7
	RSV A internal control	P1				46		
		P2				64		
		P3				86		
RSV A clinical samples from ADH	ADH RSV A sample 1-8	P1	100	100	100	17,404,684	33,988,945	322842.95
		P1	100	100	100	237,562	217,966	2074.22
		P1				51,186		
		P1	100	100	100	17,478,648	30,962,849	294822.21
		P1	100	100	100	201,554	86,234	818.34
		P1	100	100	100	4,699,770	4,618,529	43882.36
		P1	100	100	100	18,380,514	24,433,790	228064.64
		P1	98	97	92	56,034	20,002	186.07
	replicates for 1,2,6,7,8 from above	P3	100	100	100	9,432,038	9,082,853	84828.4
		P3	100	100	99	153,244	139,668	1303.2
		P3	100	100	100	3,442,772	3,353,843	31324.2
		P3	100	100	100	3,249,852	3,129,716	28571.1
		P3	99	97	92	34,334	11,382	105.5
	ADH RSV A sample 9-23	P4				44,556		
		P4	100	100	99	147,388	113,806	1,020
		P4	97	77	42	4,586	3,810	33
		P4	100	100	99	293,122	283,774	2,594
		P4	74	4	0	18,038	332	3
		P4	100	100	100	4,182,804	4,148,863	37,973
		P4	100	98	91	39,208	38,566	336
		P4	100	100	99	1,577,516	180,025	1,616
		P4	100	100	100	431,310	428,941	3,834
		P4	98	61	4×10^{-1}	1,798,582	1,275	12
		P4	100	100	100	10,412,864	10,115,482	90,680
		P4	100	98	90	19,084	10,186	95
		P4	100	100	100	10,804,060	10,427,541	97,562
		P4	100	100	100	338,634	201,996	1,881
		P4	100	100	99	145,748	138,942	1,298
Synthetic RSV B RNA	20 K	P1	100	100	100	1,707,604	1,648,391	15278.86
		P2	100	100	100	5,639,206	5,456,832	50441.77
	2K	P1	100	100	100	1,448,340	1,394,206	12884.31
		P2	100	100	100	2,161,696	2,092,043	19310.32
	200	P1	100	100	100	766,932	738,874	6826.28
		P2	100	100	100	1,887,946	1,823,286	16825.80
RSV B Internal control		P1				34		
		P2				148		
RSV B clinical samples from ADH	ADH RSV B	P1	100	100	100	2,793,316	2,622,644	24943.25
	Replication from above	P3	100	100	100	2,282,996	1,952,189	18110.2
	ADH RSV sample 2-4	P3	99	94	88	2,449,542	12,058	113.5
		P3	100	100	100	7,756,688	7,514,414	70501.0
		P3	100	100	100	2,446,858	167,519	1574.8
	ADH RSV B sample 5-15	P4	1			42,248	2	0
		P4	100	100	100	3,348,568	3,327,713	28,921
		P4	100	98	96	77,612	76,597	631
		P4	100	100	100	1,179,328	1,147,840	10,244
		P4	1			300,960	2	0
		P4	97	54	1	158,076	1,269	11
		P4	99	92	55	354,970	3,549	32
		P4	100	100	100	184,532	175,577	1,612
		P4	22			9,244	44	0
		P4	100	100	100	1,675,072	1,656,271	15,289
		P4	0			430		

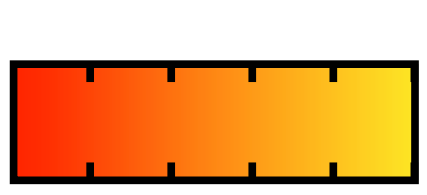

Fig. S10: Validation of synthetic and clinical RSV A and B samples

High-quality paired-end reads (minimum read length ≥70 bp) were mapped to a panel of RSV A and B reference genomes using minimap2. The mapping results using reference strains RSVA_EPI_ISL_412866 and RSVB_EPI_ISL_1653999 are shown here. Mapping results are shown for both synthetic and clinical samples at 1×, 10×, and 30× depth. The number of high-quality forward (R1) and reverse (R2) reads, mapped reads, and mean coverage are summarised in adjacent tables. Genome coverage proportions were calculated with Qualimap and visualised as heat maps. Abbreviations: P1–P4 = 96-well production plates 1–4; ADH = Addenbrooke's Hospital, Cambridge; RSV = respiratory syncytial virus.
