# Supplementary material for: Benchmarking and optimisation of bait-capture metagenomics for sequencing of respiratory viruses at scale
Source: Genome Med. 2026 Jul 30;18:166. doi: 10.1186/s13073-026-01725-8 (PMC13715170; doi:10.1186/s13073-026-01725-8)
Supplement: Supplementary file 4 [file 13073_2026_1725_MOESM2_ESM.pdf]

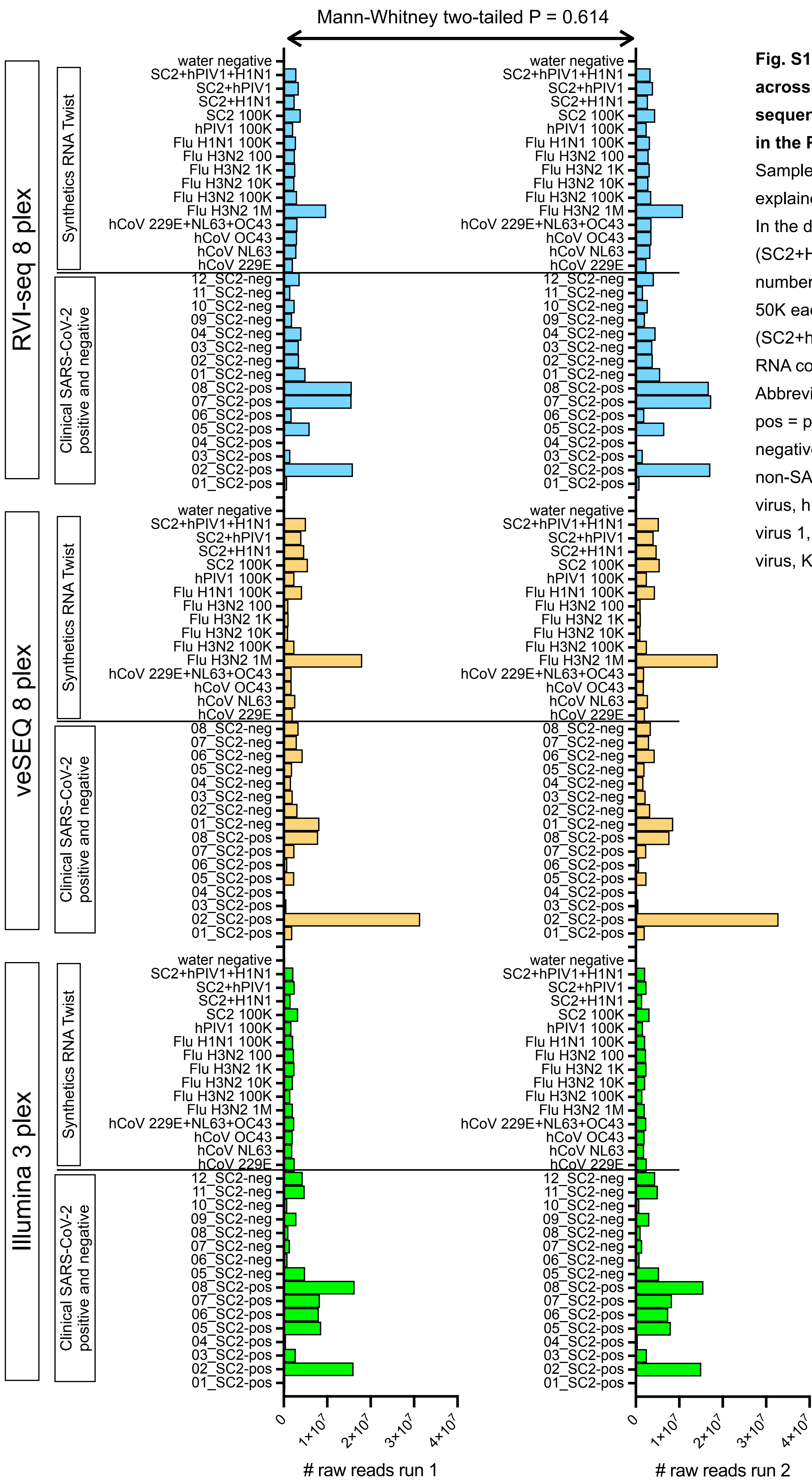

Fig. S1: Number of raw reads across the two replicated sequencing runs were very similar in the Pilot 1 experiment
Sample types and sequence IDs are explained in **Extended Data Table 1**. In the double mix samples (SC2+H1N1 and SC2+hPIV1), the number of RNA copies added was 50K each, and in the triple mix sample (SC2+hPIV1+H1N1), the number of RNA copies added was 33K each. Abbreviations: SC2 = SARS-CoV-2, pos = positive for SARS-CoV-2, neg = negative for SARS-CoV-2, hCoV = non-SARS-CoV-2 human corona virus, hPIV = human parainfluenza virus 1, H3N2 and H1N1 = influenza A virus, K = thousand
