# Supplementary material for: Benchmarking and optimisation of bait-capture metagenomics for sequencing of respiratory viruses at scale
Source: Genome Med. 2026 Jul 30;18:166. doi: 10.1186/s13073-026-01725-8 (PMC13715170; doi:10.1186/s13073-026-01725-8)
Supplement: Supplementary file 5 [file 13073_2026_1725_MOESM3_ESM.pdf]

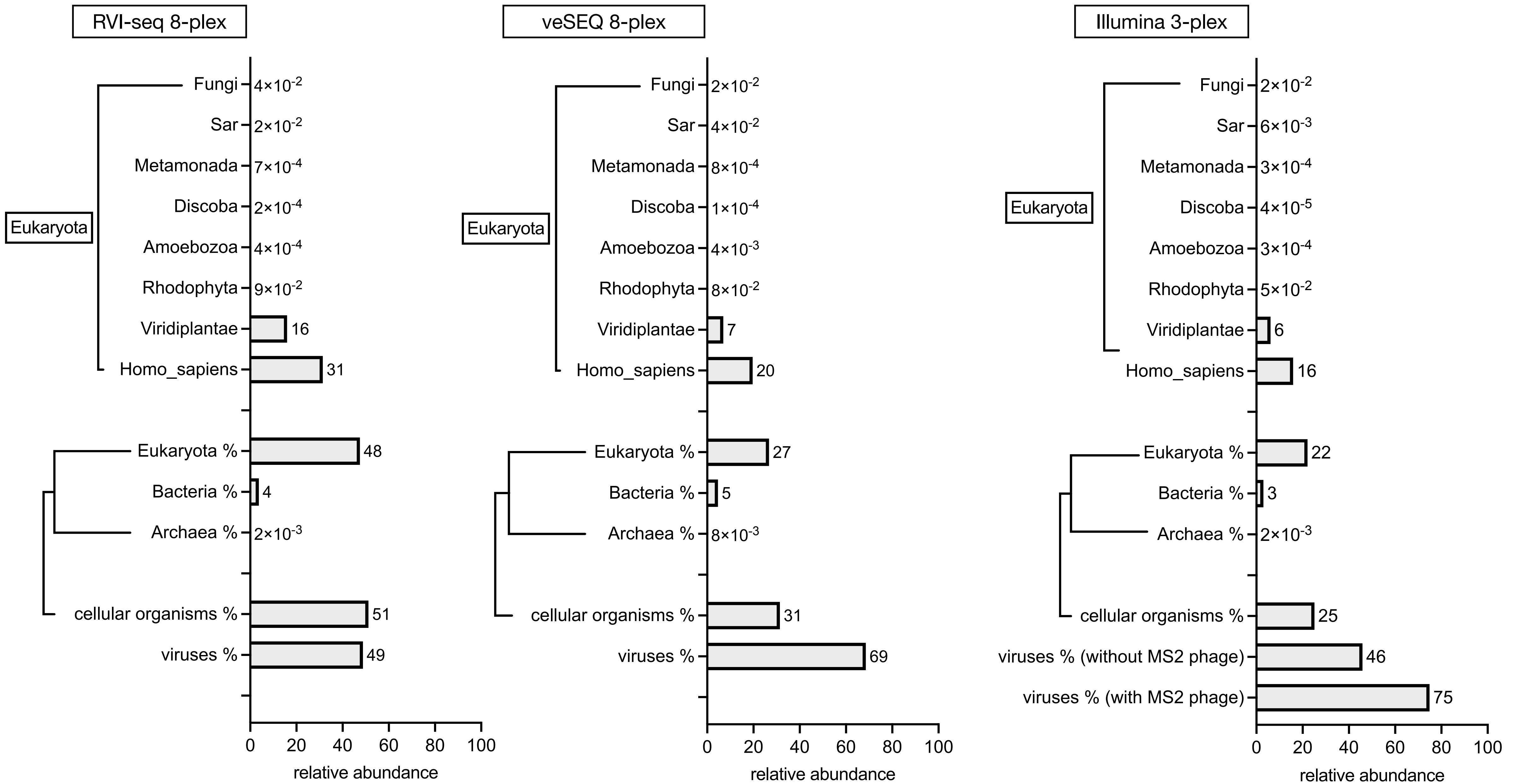

Fig. S2: Higher classification differences detected between the three bait capture methods in the Pilot 1 study

The Kraken PlusPFP database (standard plus RefSeq protozoa, fungi and plants, updated 14/03/2023) was used to classify the RVI Pilot 1 study (first run) at the highest taxonomic level. Relative abundances for viruses and cellular organisms are shown in a bar chart. In the Illumina analysis, the proportional abundances of the viruses are shown with and without spiked MS2 phage for better comparison with the virus proportions of the RV-seq and veSEQ methods which were prepared without MS2 spiked phage. The cellular organisms were then further classified into eukaryotes, bacteria, and archaea. Finally, the eukaryotes were subdivided further into Homo sapiens and others. The relative proportion of taxonomic composition was calculated from the total number of Kraken2 hits using Microsoft Excel for Mac (version 16.72). The plot was generated in Prism 9 for macOS (v. 9.5.1) and finalised in Keynote for Mac (v. 13.0).
