# Supplementary material for: Benchmarking and optimisation of bait-capture metagenomics for sequencing of respiratory viruses at scale
Source: Genome Med. 2026 Jul 30;18:166. doi: 10.1186/s13073-026-01725-8 (PMC13715170; doi:10.1186/s13073-026-01725-8)
Supplement: Supplementary file 6 [file 13073_2026_1725_MOESM4_ESM.pdf]

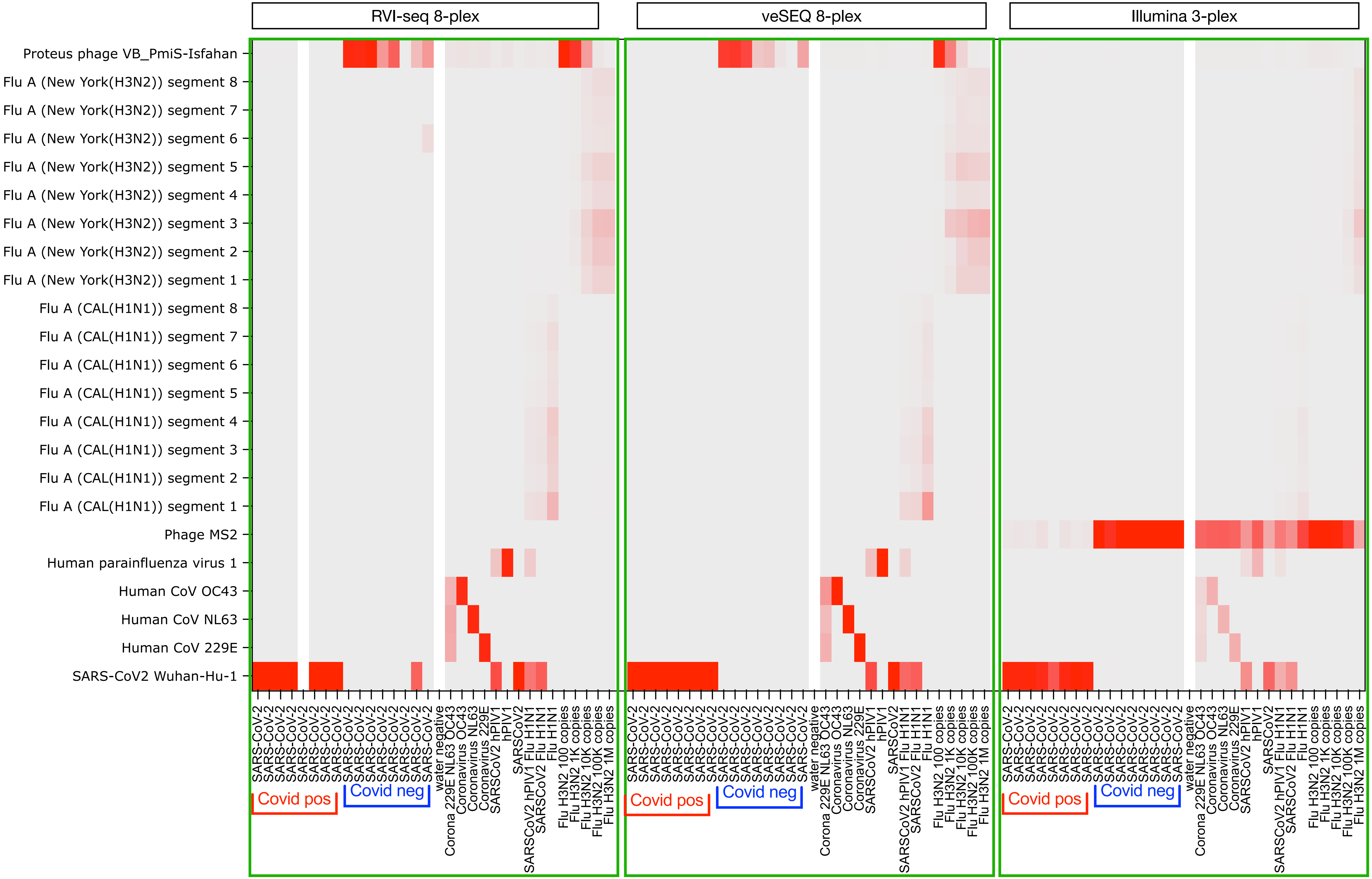

Fig. S3: Pilot 1 - Viral Bait Capture inStrain analysis demonstrating the high abundance of spiked-in phage MS2 in the Illumina dataset

Paired end reads of a minimum length of 100 bp were used for taxonomic classification with inStrain. Only viral taxa n=22/61 with a minimum abundance of 0.07% are presented; this encompasses 99.93% of all viral hits. The strong signal of the spike phage MS2 in the Illumina dataset accounted for a large proportion of sequences, which resulted in a markedly weaker signal for the influenza A virus and human seasonal coronavirus. The clinical positive SARS-CoV-2 samples exhibited less impact from the spike MS2 phage. Due to the impact of the MS2 spike on viral signal strength and to standardise with the AMPHEUS and RVI-seq methods, we did not spike MS2 into any further experiments.
