# Supplementary material for: Benchmarking and optimisation of bait-capture metagenomics for sequencing of respiratory viruses at scale
Source: Genome Med. 2026 Jul 30;18:166. doi: 10.1186/s13073-026-01725-8 (PMC13715170; doi:10.1186/s13073-026-01725-8)
Supplement: Supplementary file 7 [file 13073_2026_1725_MOESM5_ESM.pdf]

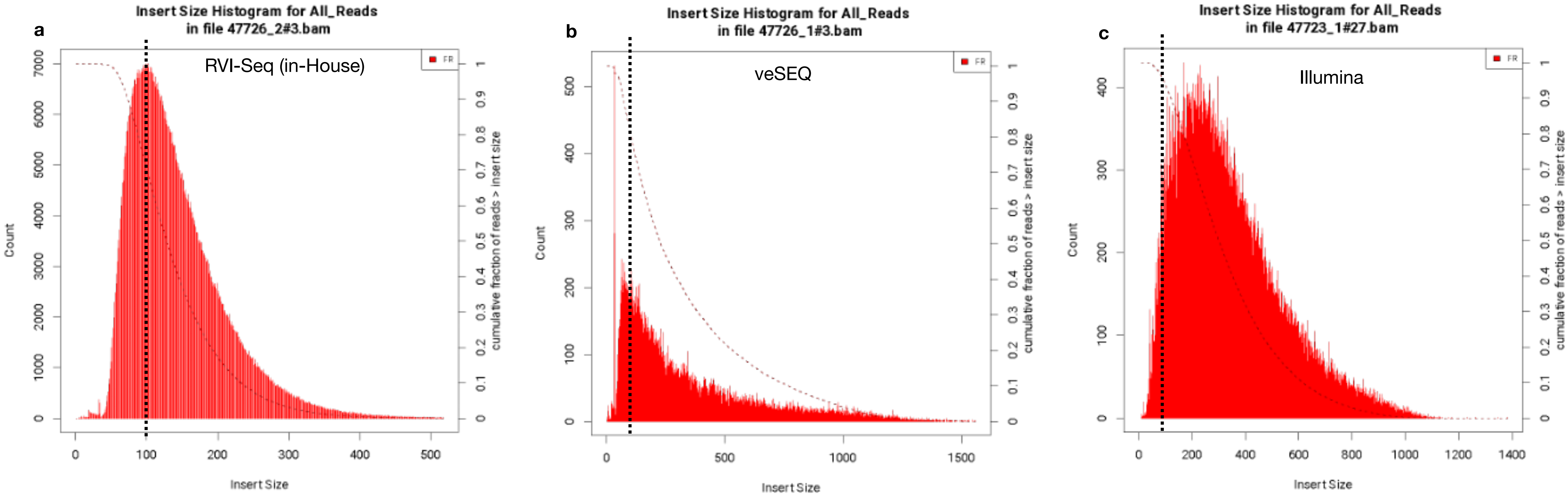

Fig. S4: Insert size distribution is smallest in the RVI-seq bait capture method

The insert size distribution for a Twist synthetic SARS-CoV-2 sample (100k copies) is shown, from which the largest proportion of reads below 100bp length were removed by Kneaddata filtering from the inHouse method (**a**), compared to the Ampheus method (**b**) and the Illumina method (**c**).
