# Supplementary material for: Benchmarking and optimisation of bait-capture metagenomics for sequencing of respiratory viruses at scale
Source: Genome Med. 2026 Jul 30;18:166. doi: 10.1186/s13073-026-01725-8 (PMC13715170; doi:10.1186/s13073-026-01725-8)
Supplement: Supplementary file 8 [file 13073_2026_1725_MOESM6_ESM.pdf]

SUPPLEMENTARY RESULTS

1 – SUPPLEMENTARY RESULTS for de-novo assembly

Supplementary Result figures 1

2 – SUPPLMENTARY RESULTS for inStrain optimisation

Supplementary Result Figures 2 to 3

1 – SUPPLEMENTARY RESULTS: De-novo Assembly

One aim of this study was to perform de-novo assembly of viral bait capture sequence data, to assess whether high-quality contigs and genomes could be assembled.

For the de-novo assembly, we used the MetaSPAdes function of SPAdes (v3.14) to assemble high-quality (HQ), short, paired end reads (minimum 100 bp) that had been filtered using KneadData. The quality of the resulting MetaSPAdes contigs and genomes was assessed using the MetaQUAST function of QUAST (v4.4). Several metrics were analysed using MetaQUAST, shown here are the total number of contigs generated at various minimum length thresholds: no minimum length, 1 kb, 5 kb, 10 kb, and 25 kb, for the three viral BCMs in the Pilot 1 study (see **Supplementary Result Figure 1**). In Pilot 1, three viral BCMs were compared: veSEQ 8-plex, RVI-seq 8-plex (in-house), and Illumina 3-plex.

Contig numbers across different viral BCMS (Supplementary Result Figure 1)

De-novo assembly using MetaSPAdes revealed significant differences in the total number of contigs generated across the three distinct viral BCMs. When contig numbers per sample were compared between the three BCMs using a non-parametric paired Friedman test with Dunn's multiple comparisons adjustment, significant differences were observed. (**a**) Specifically, the comparison between the RVI-seq and veSEQ methods ($p = 0.009$) and between the veSEQ and Illumina methods ($p = 0.0295$) were statistically significant for all contigs. (**b**) For the ≥1 kb contig dataset, the only significant difference was found between the RVI-seq and Illumina methods ($p = 0.0201$). (**c**) No significant differences were detected between the three BCMs for the ≥5 kb contig dataset. (**d**) Notably, the veSEQ and RVI-seq methods produced the highest number of contigs ≥25 kb for six of the eight Heron-positive COVID-19 samples, while the Illumina method generated only two contigs corresponding to the SARS-CoV-2 genome.

2 – SUPPLEMENTARY RESULT for inStrain optimisation

Another objective of this study was to optimize inStrain analysis for the taxonomic classification of viral bait captured short reads by comparing three different analysis options in inStrain. The optimal inStrain configuration was then compared with Kraken2/Bracken analysis. Initial optimization was conducted on sequence data from the ZeptoMetrix Respiratory Panel 2.1 (RP2.1) controls, followed by a subsequent test on high-quality paired-end short reads (down sampled to a maximum of 1 million reads) from the Pilot 2 viral BCMs. Three distinct inStrain options were evaluated: the first option utilized database mode with a minimum genome coverage of 1 and a minimum read average nucleotide identity (ANI) of 0.92; the second option did not employ database mode, set genome coverage to zero, and had a minimum read ANI of 0.9; the third option, the default setting, also did not use database mode, but had a minimum genome coverage of zero and a minimum read ANI of 0.95. inStrain (option 2) produced the most accurate mapping results and was therefore selected for comparison with Kraken2/Bracken. In an initial analysis, we compared viral classifications obtained from the ZeptoMetrix control samples, **Supplementary Result Figure 2**. This comparison revealed that both inStrain and Kraken2/Bracken analysis yielded similar results. However, Kraken2 failed to detect RSV A, while inStrain successfully identified RSV A across all four bait capture methods. Additionally, Kraken2 was unable to distinguish between different influenza A strains and did not identify signals for the various influenza strain segments. Among the four bait capture methods, the veSEQ method failed to detect certain viruses present in ZeptoMetrix RP2.1 Control 2, which were successfully identified by the RVI-seq method and Illumina method (at both 3-plex and 8-plex). In a second comparison we compared NIBSC flu samples, and parainfluenza, metapneumovirus, rhinovirus, and their mixtures, as well as nasal swab negative samples with and without spike-ins containing either H1N1, H3N2, RSV B, FLU B, or SARS-CoV-2 (**Supplementary Result Figure 3**).

The results for influenza A, influenza B, and RSV B are presented in **Supplementary Result Figure 3a**. Both Kraken and inStrain option 2 were able to detect influenza B at a copy number as low as 1k. In the 10k copy number sample, inStrain option 2 detected four segments, option 1 detected two segments, and option 3 did not detect any segments. Samples processed using the veSEQ and RVI-seq methods produced the most consistent and accurate results.

The RVI-seq and veSEQ methods were the most effective at detecting individual influenza A H3N2 segments (highlighted by black boxes). In the 100-copy number sample, the RVI-seq method identified two segments, with inStrain options 2 and 3. None of the Illumina methods were able to detect a signal in the 100-copy number sample. The RVI-seq method also successfully detected all eight segments in the 1k copy number sample, regardless of

the inStrain option used. All segments were detectable in the 10k, 100k, and 1M copy number samples across all methods.
For influenza A H1N1 segments (highlighted in red boxes), inStrain options 2 and 3, along with the RVI-seq method, were the most effective, particularly at the 100 and 1k copy number samples. However, the enhanced sensitivity of inStrain options 2 and 3 slightly reduced specificity, leading to weak, non-specific detections between different influenza strains.
All four viral BCMs methods detected the influenza A virus (H3N2 and H1N1) in the 1k copy number sample using Kraken. However, only the RVI-seq method detected signals from the 100-copy number sample for both H3N2 and H1N1 (Kraken column).
For RSV B, both Kraken and inStrain methods classified the virus as Human Orthopneumovirus. The veSEQ and Illumina methods detected the virus at 1k sample using Kraken and all three inStrain options. Additionally, the RVI-seq method successfully detected the virus at 100 copies per sample, but only with Kraken and inStrain options 2 and 3 (highlighted by blue arrows).
Kraken identified Alphapapillomavirus 7, while inStrain detected human papillomavirus in most samples, with varying signal intensities. The signal was particularly prominent in the low-copy number samples, suggesting potential contamination from papillomavirus, especially in low-biomass samples. Kraken identified Human Mastadenovirus B, while inStrain detected Human Adenovirus B1 as contaminants in the RSV B negative control sample.

The last set of samples are shown in **Supplementary Result Figure 3b.** All four bait capture methods and BCMs successfully detected human metapneumovirus. Detection of human rhinovirus A, either alone or in combination with H1N1, was only possible through the identification of a weak signal for rhinovirus C using Kraken. This was observed exclusively in samples from the RVI-seq BCM (indicated by red arrows). In the multiplex sample containing influenza, RSV, and SARS-CoV-2, only the SARS-CoV-2 signal was detected across all four analytical methods and BCMs.

In the five nasal swab negative samples, the veSEQ method produced no signal, while the RVI-seq method generated only weak signals (shown in black boxes). However, when using the two Illumina bait capture methods, all four analytical methods detected robust signals for Human Orthopneumovirus and Human Respirovirus 1/Human Parainfluenza Virus (indicated by black boxes).

Kraken successfully identified both H1N1 and H3N2 in the negative nasal swab samples spiked with the two influenza strains. However, inStrain options 2 and 3 were only able to detect all eight segments for both strains in the samples prepared with the RVI-seq and Illumina methods (depicted by the black dashed ellipse). The veSEQ method failed to detect certain segments.

In conjunction with Kraken and inStrain, as well as the veSEQ, RVI-seq, and Illumina 3-plex methods, the presence of respiratory syncytial virus (RSV B), also known as human Orthopneumovirus, was identified in the third nasal swab sample spiked with RSV B.

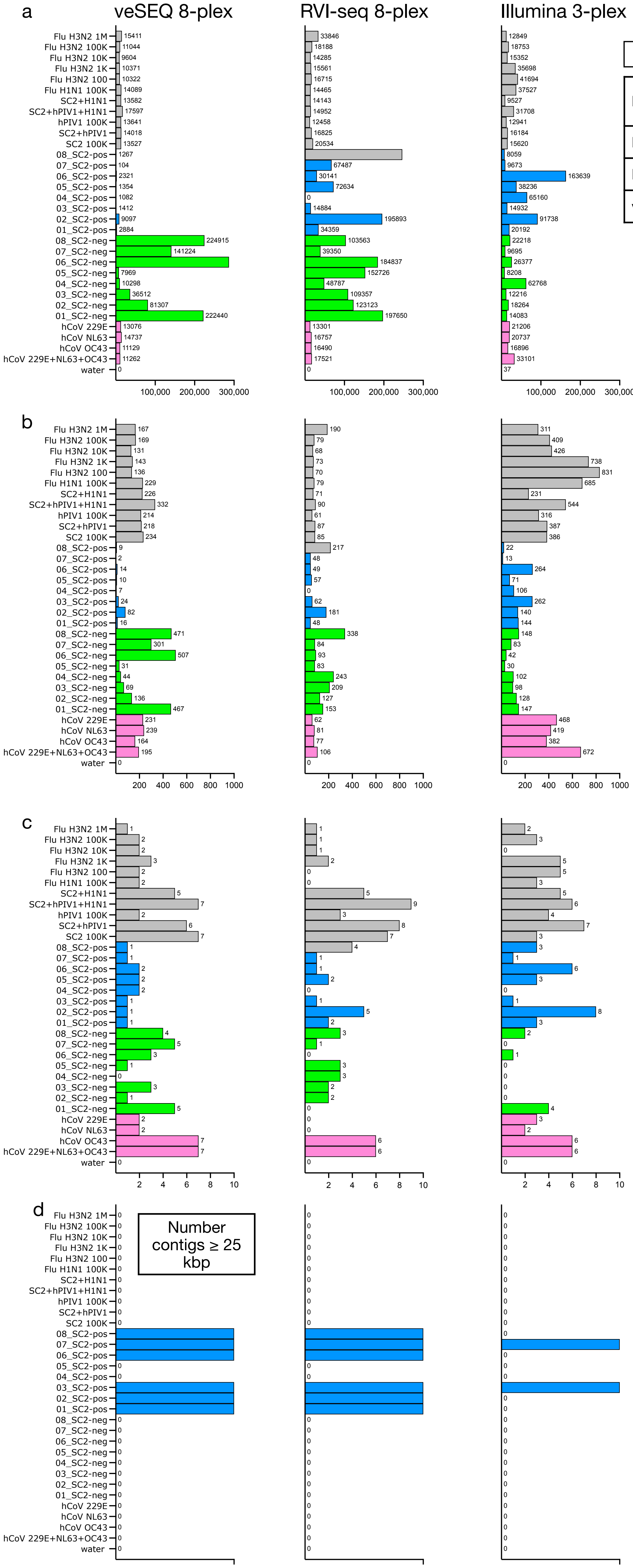

Number contigs ≥ 0 bp

Dunn's multiple comparisons test	Rank sum diff.	Adjusted P Value
RVI-seq# contigs (≥0kb) vs. veSEQ# contigs (≥0kb)	28.00	0.0009
RVI-seq# contigs (≥0kb) vs. Ill# contigs (≥0kb)	8.000	0.9051
veSEQ# contigs (≥0kb) vs. Ill# contigs (≥0kb)	-20.00	0.0295

Number contigs ≥ 1 kbp

Dunn's multiple comparisons test	Rank sum diff.	Adjusted P Value
RVI-seq# contigs (≥1kb) vs. veSEQ# contigs (≥1kb)	-3.000	>0.9999
RVI-seq# contigs (≥1kb) vs. Ill# contigs (≥1kb)	-21.00	0.0201
veeSEQ# contigs (≥1kb) vs. Ill# contigs (≥1kb)	-18.00	0.0604

Number contigs ≥ 5 kbp

Dunn's multiple comparisons test	Rank sum diff.	Adjusted P Value
RVI-seq# contigs (≥5kb) vs. veSEQ# contigs (≥5kb)	-7.000	>0.9999
RVI-seq# contigs (≥5kb) vs. Ill# contigs (≥5kb)	-8.000	0.9051
veSEQ# contigs (≥5kb) vs. Ill# contigs (≥5kb)	-1.000	>0.9999

Supplementary Result Figure 1: Number of contigs differs between bait capture methods in Pilot 1 study
HQ paired-end short reads with a minimum length of 100bp were de-novo assembled using MetaSPAdes and the quality of the contigs was assessed using MetaQuast. The total number of contigs was determined with no minimum length (a), with a minimum length of 1kb (b), 5kb (c) and 25kb (d). Clinical positve and negative SARS-CoV-2 are coloured with blue and green, respectively. Non SARS-CoV-2 human corona virus are colour coded with pink. The number of contigs is shown on the x-axis, with some differences noted between the three different bait capture methods. Abbreviations: hCoV = non SARS-CoV-2 human coronavirus, SC2 = SARS-CoV-2, neg = negative, pos = positive, hPIV1 = human parainfluenza virus 1, 1M, 100K, 10K, 1K = number of Twist Synthetic RNA copies used as Input into workflow, RVI-seq (inHouse), veSEQ, and Ill are the different bait capture methods used in pilot 1 study

a. inStrain option2

ZeptoMetrix taxa		ZeptoMetrix RP 2.1 Control 1				ZeptoMetrix RP 2.1 Control 2			
		veSEQ	RVI-seq	Il 3pl	Il 8pl	veSEQ	RVI-seq	Il 3pl	Il 8pl
Adenovirus Type 1	Human adenovirus 1	0.311	0.076	1.570	1.476				0.001
	Human adenovirus 2	0.015	0.005	0.089	0.085				
	Human adenovirus 5	0.012	0.003	0.081	0.072				
	Human adenovirus 7	24.527	23.755	23.541	23.139		0.001	0.001	0.001
	Human adenovirus A	0.021	0.002	0.001	0.001				
Adenovirus Type 3	Human mastadenovirus A	0.017	0.003		0.001				
	Human adenovirus B1	71.073	68.528	71.645	72.095		0.001	0.003	0.006
	Human adenovirus B2	0.008	0.114	0.038	0.045				
	Human adenovirus C	0.010	0.005	0.090	0.091				
	Human adenovirus E			0.001					
Adenovirus Type 31	Human adenovirus type 35	0.025	0.078	0.022	0.020				
Influenza A H1N1 pandemic A/NY/ 02/2009	FLU A (H1N1 CAL) sgm 1	0.420	0.936	0.355	0.374	0.015	0.019	0.009	0.006
	FLU A (H1N1 CAL) sgm 2	0.443	1.338	0.385	0.422				
	FLU A (H1N1 CAL) sgm 3	0.461	0.884	0.242	0.266				
	FLU A (H1N1 CAL) sgm 4	0.376	0.752	0.290	0.284				
	FLU A (H1N1 CAL) sgm 5	0.312	0.649	0.317	0.327				
	FLU A (H1N1 CAL) sgm 6	0.211	0.542	0.201	0.208				
	FLU A (H1N1 CAL) sgm 7	0.293	0.483	0.208	0.197				
	FLU A (H1N1 CAL) sgm 8	0.187	0.357	0.106	0.113				
Influenza A H3N2 A/ Brisbane/ 10/07	FLU A (H3N2 NY) sgm 1					0.015	0.009	0.002	0.001
	FLU A (H3N2 NY) sgm 2	0.011	0.012	0.001					
	FLU A (H3N2 NY) sgm 3					0.008	0.001		0.001
	FLU A (H3N2 NY) sgm 4	0.091	0.142	0.060	0.061				
	FLU A (H3N2 NY) sgm 5								0.001
	FLU A (H3N2 NY) sgm 6	0.040	0.069	0.037	0.036				
	FLU A (H3N2 NY) sgm 7					0.030	0.008	0.002	0.002
	FLU A (H3N2 NY) sgm 8								0.004
Metapneumonia virus 8	Human metapneumovirus		0.004						
Parainfluenza Type 1	Human parainfluenza 1	0.063	0.160	0.071	0.073				
Parainfluenza Type 4	Human parainfluenza 4a	0.072	0.333	0.128	0.125				
Rhinovirus 1A	Human rhinovirus 1	0.116	0.098	0.002	0.002				
	SARS-Cov2	0.044	0.115	0.103	0.054			0.001	
Influenza A H1N1 A/ New Caledonia / 20/1999	FLU A (H1N1 PRI) sgm 1	0.327	0.141	0.083	0.078	2.445	0.258	0.100	0.116
	FLU A (H1N1 PRI) sgm 2	0.079	0.056	0.121	0.151	0.218	0.030	0.118	0.116
	FLU A (H1N1 PRI) sgm 3	0.076	0.093	0.089	0.074	0.248	0.169	0.042	0.046
	FLU A (H1N1 PRI) sgm 4					0.008	0.010	0.055	0.060
	FLU A (H1N1 PRI) sgm 5	0.097	0.080	0.064	0.075	0.376	0.142	0.018	0.027
	FLU A (H1N1 PRI) sgm 6						0.002	0.019	0.015
	FLU A (H1N1 PRI) sgm 7	0.126	0.123	0.019	0.021	1.753	0.351	0.098	0.096
	FLU A (H1N1 PRI) sgm 8	0.038	0.039	0.016	0.010	0.098	0.103	0.010	0.012
	Human orthopneumovirus A						0.013	0.006	0.007
Coronavirus 229E	Human coronavirus 229E					20.632	36.534	40.995	39.913
Coronavirus HKU1	Human coronavirus HKU1						0.012	0.005	0.006
CoronavirusNL43	Human Coronavirus NL63					1.565	3.214	3.111	3.085
Coronavirus OC43	Human coronavirus OC43					7.698	8.087	10.348	10.136
Influenza B B/Florida 02/06	FLU B sgm 1					4.560	5.066	3.844	4.001
	FLU B sgm 2					5.455	5.192	4.522	4.828
	FLU B sgm 3					6.825	4.949	3.179	3.490
	FLU B sgm 4					1.535	2.430	1.730	1.841
	FLU B sgm 5					3.567	3.898	3.197	3.261
	FLU B sgm 6					0.873	1.769	1.456	1.578
	FLU B sgm 7					1.836	1.818	1.429	1.506
	FLU B sgm 8					2.092	2.511	2.163	2.220
Parainfluenza Type 3	Human parainfluenza 3						0.005	0.001	0.001
	Human parainfluenza 3					0.339	0.166	0.061	0.058
Parainfluenza Type 2	Human rubulavirus 2					29.022	20.565	22.711	22.760
RSV A	Respiratory syncytial virus					0.632	0.142	0.099	0.114

b. Kraken2/Bracken

ZeptoMetrix taxa		ZeptoMetrix RP 2.1 Control 1				ZeptoMetrix RP 2.1 Control 2			
		veSEQ 8plex	RVI-seq 8plex	Illumina 3plex	Illumina 8plex	veSEQ 8plex	RVI-seq 8plex	Illumina 3plex	Illumina 8plex
Adenovirus Type 1	Human mastadenovirus A	0.026	0.003	0.001	0.002				
Adenovirus Type 3	Human mastadenovirus B	95.258	92.243	94.988	95.170		0.002	0.004	0.007
Adenovirus Type 31	Human mastadenovirus C	0.512	0.136	2.300	2.105			0.001	0.001
	Human mastadenovirus E	0.001	0.001	0.001	0.002				
Influenza A 2009 H1N1	Influenza A virus	3.986	7.215	2.533	2.584	14.425	4.308	1.193	1.271
Metapneumonia virus 8	Human metapneumovirus	0.015	0.002	0.001	0.001				
Parainfluenza Type 1	Human respirovirus 1	0.030	0.082	0.034	0.038				
Parainfluenza Type 4	Human orthorubulavirus 4	0.035	0.124	0.045	0.051				
Rhinovirus 1A	Rhinovirus A	0.100	0.096	0.003	0.002				
	SARS-CoV2	0.037	0.097	0.095	0.045				
	Bovine orthopneumovirus					0.354	0.118	0.097	0.104
	Human orthopneumovirus					0.066	0.035	0.016	0.018
Coronavirus 229E	Human coronavirus 229E					20.285	37.150	42.496	40.462
Coronavirus HKU1	Human coronavirus HKU1						0.012	0.013	0.014
CoronavirusNL43	Human coronavirus NL63					1.524	3.170	3.060	2.987
Coronavirus OC43	Betacoronavirus 1					7.415	8.066	9.854	9.540
Influenza B	Influenza B virus					24.982	26.349	20.667	23.303
Parainfluenza Type 2	Human orthorubulavirus 2					30.693	20.561	22.505	22.204
Parainfluenza Type 3	Human respirovirus 3					0.257	0.229	0.092	0.089

Supplementary Result Figure 2: ZeptoMetrix RP 2.1 sequencing and classification comparison between inStrain and Kraken2/Bracken

inStrain with option 2 parameters (no database mode, min genome coverage 0, min read ani of 0.9) compared with Kraken2/Bracken analysis using HQ paired end short read sequences from the ZeptoMetrix Respiratory Virus Control 1 and 2. The relative abundances of the identified viral signals are shown in a heat map generated in GraphPad Prism (v 10.0.3). The ZeptoMetrix taxa are shown in boxes and single lines under the heading 'ZeptoMetrix taxa'. The Zepto Metrix control was sequenced using the same viral bait capture methods used in the Pilot 2 experiment.

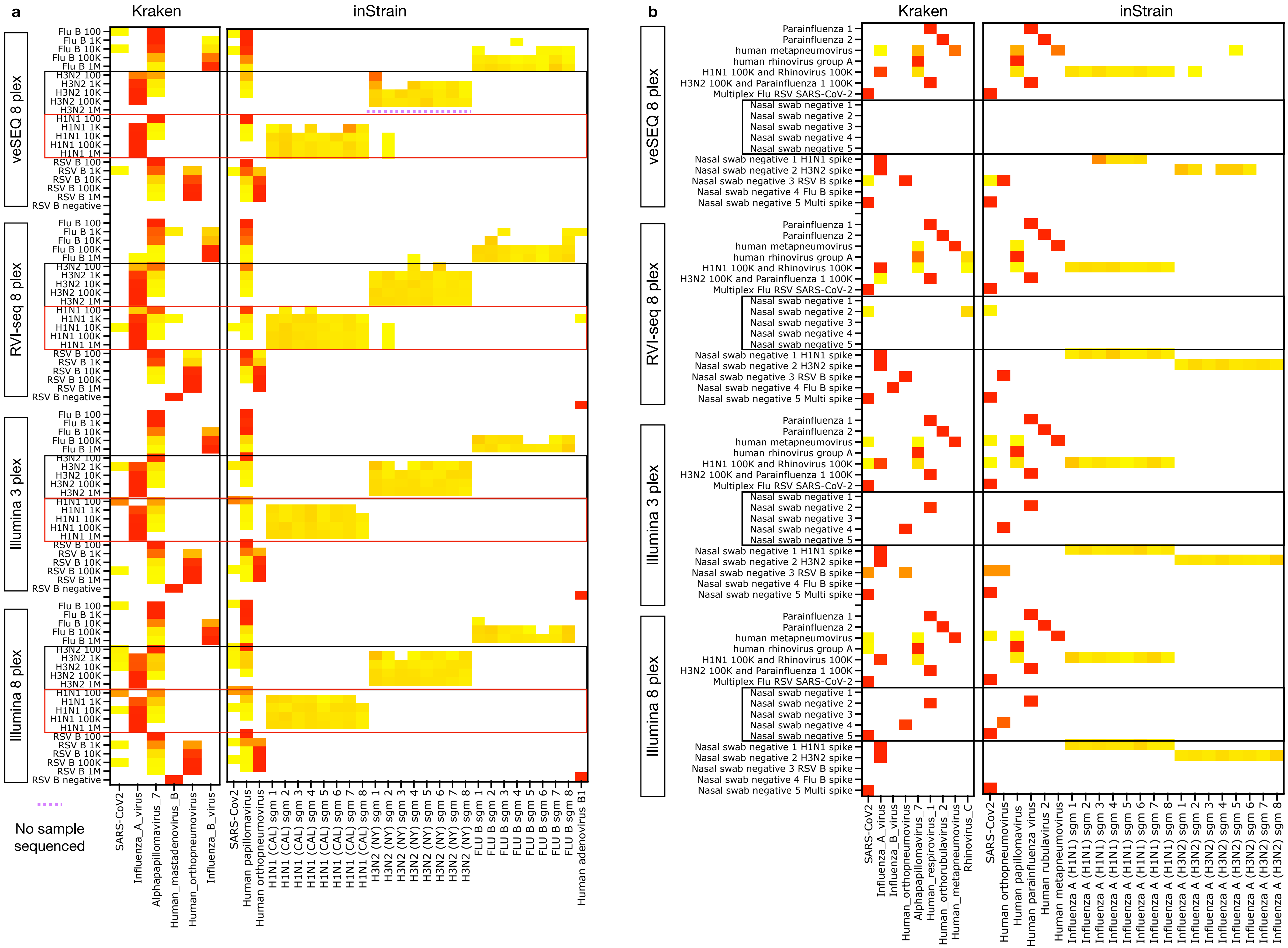

Supplementary Result Figure 3: NIBSC sample comparison between Kraken and inStrain

High-quality paired-end short reads were downsampled to a maximum of 1 million reads and taxonomically classified using Kraken2/Bracken and inStrain (option 2 parameters). Only taxa with a minimum of 1,000 reads and a relative abundance ≥2% in at least one sample are shown. Samples include: (a) dilution series for FluB, H3N2, H1N1, and RSV; (b) parainfluenza virus, metapneumovirus, rhinovirus, and their mixtures, and nasal swab negative samples with and without viral spike-ins.
