# Supplementary material for: Benchmarking and optimisation of bait-capture metagenomics for sequencing of respiratory viruses at scale
Source: Genome Med. 2026 Jul 30;18:166. doi: 10.1186/s13073-026-01725-8 (PMC13715170; doi:10.1186/s13073-026-01725-8)
Supplement: Supplementary file 9 [file 13073_2026_1725_MOESM7_ESM.pdf]

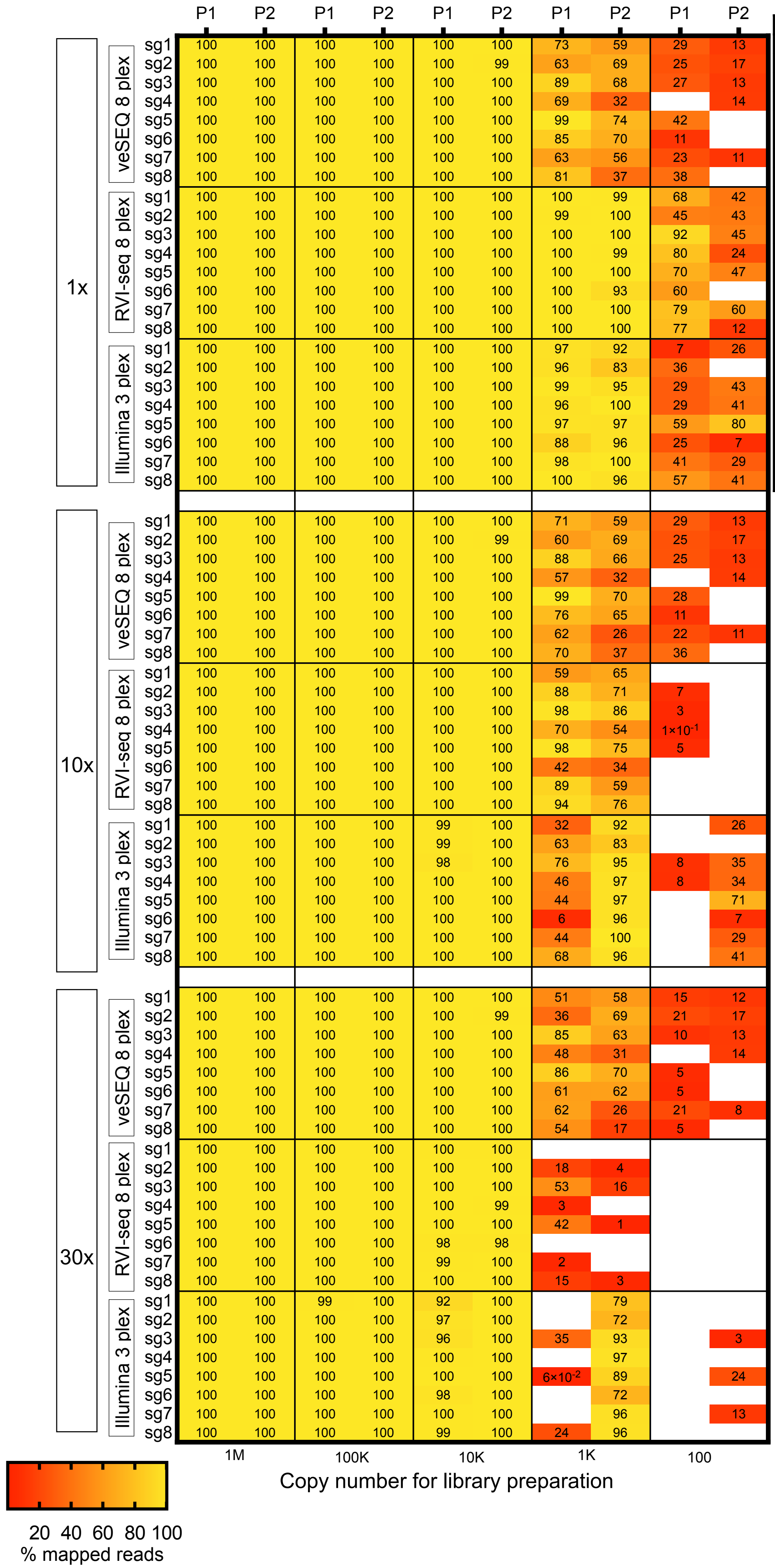

# mapped reads used for reference mapping: 1M		100K		10K		1K		100		Mean coverage: 1M		100K		10K		1K		100	
Pilot1	Pilot2	Pilot1	Pilot2	Pilot1	Pilot2	Pilot1	Pilot2	Pilot1	Pilot2	Pilot1	Pilot2	Pilot1	Pilot2	Pilot1	Pilot2	Pilot1	Pilot2	Pilot1	Pilot2
3,376,205	293,290	278,487	281,591	9,173	17,205	1,206	2,433	156	216	186,571	16,741	15,550	16,152	523	977	62	144	8	12
4,018,345	291,055	330,107	332,382	10,387	20,734	959	2,337	174	254	222,101	16,616	18,304	18,918	566	1,167	51	136	10	16
8,800,497	684,126	690,447	696,086	25,166	52,291	4,700	4,163	214	230	512,804	40,874	40,259	41,450	1,449	3,094	277	243	12	12
1,731,063	144,251	152,183	127,024	4,908	11,065	798	660		250	130,824	11,157	11,507	9,812	368	854	58	51		18
3,064,091	237,959	268,963	220,299	10,525	19,362	1,469	1,282	98		262,156	20,818	23,120	19,376	887	1,724	127	118	7	
1,287,975	89,948	110,116	90,749	4,070	7,623	644	1,370	37		119,343	8,514	10,202	8,587	373	719	60	133	3	
1,900,666	144,147	166,293	144,485	5,851	12,291	636	266	120	64	244,584	18,915	21,366	19,050	755	1,628	76	30	16	5
1,739,864	111,775	138,894	105,625	3,725	6,964	582	227	52		250,513	16,731	20,208	15,853	558	1,061	84	34	7	
1,166,902	270,019	89,685	49,892	3,511	3,601	231	233	28	16	63,753	14,300	4,883	2,615	184	184	11	12	1	1
1,573,524	374,779	124,637	68,493	4,651	5,155	387	301	36	27	86,108	19,843	6,779	3,588	244	262	20	15	2	1
2,095,273	493,747	164,203	92,768	5,746	6,902	545	355	62	24	120,029	27,377	9,362	5,091	315	372	29	19	3	1
799,800	187,566	61,043	34,523	2,232	2,646	194	150	28	6	58,131	13,190	4,420	2,398	159	182	14	11	2	1
1,143,987	266,270	89,533	50,543	3,436	3,931	360	194	36	15	92,826	20,864	7,205	3,905	265	295	28	14	3	1
464,968	109,289	36,854	20,646	1,458	1,471	106	86	20		40,161	9,140	3,165	1,706	121	120	9	7	2	
552,292	123,788	41,227	23,727	1,726	1,956	162	108	26	10	67,624	14,623	5,029	2,775	201	225	19	13	3	1
752,389	173,457	58,265	34,174	1,849	2,331	162	128	12	2	107,399	23,857	8,261	4,659	255	309	22	17	2	
142,478	233,307	13,181	89,026	1,406	16,655	128	912	2	72	8,665	14,362	805	5,479	85	1,017	8	56	0	5
195,717	288,932	18,443	104,351	1,659	20,272	180	1,129	8		11,966	17,848	1,129	6,442	100	1,250	11	70	0	
388,379	482,356	35,982	174,630	3,485	35,123	346	2,542	32	118	24,823	31,197	2,306	11,298	224	2,259	22	161	2	8
155,153	255,255	13,924	94,534	1,397	21,335	104	1,412	22	100	12,296	20,361	1,108	7,553	109	1,681	8	113	2	7
134,628	217,545	13,498	78,704	1,408	15,313	110	1,128	14	208	12,133	19,727	1,220	7,156	127	1,387	10	100	1	19
96,276	133,818	8,667	47,539	869	10,399	36	581	4	22	9,236	12,911	835	4,602	82	999	3	56	0	2
116,972	172,895	11,352	61,736	1,049	13,407	82	1,206	6	84	15,910	23,632	1,552	8,510	142	1,817	11	165	1	11
138,168	215,424	12,109	73,425	1,240	15,530	136	1,048	16	38	21,826	34,309	1,911	11,696	194	2,477	21	170	2	5

Fig. S5: Reference mapping for H3N2 Twist dilution series demonstrates consistent reproducibility

The reproducibility of H3N2 genome mapping was assessed across two independent experiments (P1 and P2) using three methods: veSEQ, RVI-seq, and Illumina bait capture. Samples containing 1 million to 100 RNA copies were sequenced, and the genome fraction sequenced across all eight H3N2 segments was evaluated at 1×, 10×, and 30× coverage depths. The number of reads used for mapping and the mean coverage for each condition are shown in the accompanying table.

Abbreviations: 100, 1K, 10K, 100K, 1M = input RNA copies (100, 1,000, 10,000, 100,000, 1 million); sg = H3N2 segments; 1×, 10×, 30× = sequencing depth; P1, P2 = two independent pilot experiments.
