# Supplementary material for: Benchmarking and optimisation of bait-capture metagenomics for sequencing of respiratory viruses at scale
Source: Genome Med. 2026 Jul 30;18:166. doi: 10.1186/s13073-026-01725-8 (PMC13715170; doi:10.1186/s13073-026-01725-8)
Supplement: Supplementary file 10 [file 13073_2026_1725_MOESM8_ESM.pdf]

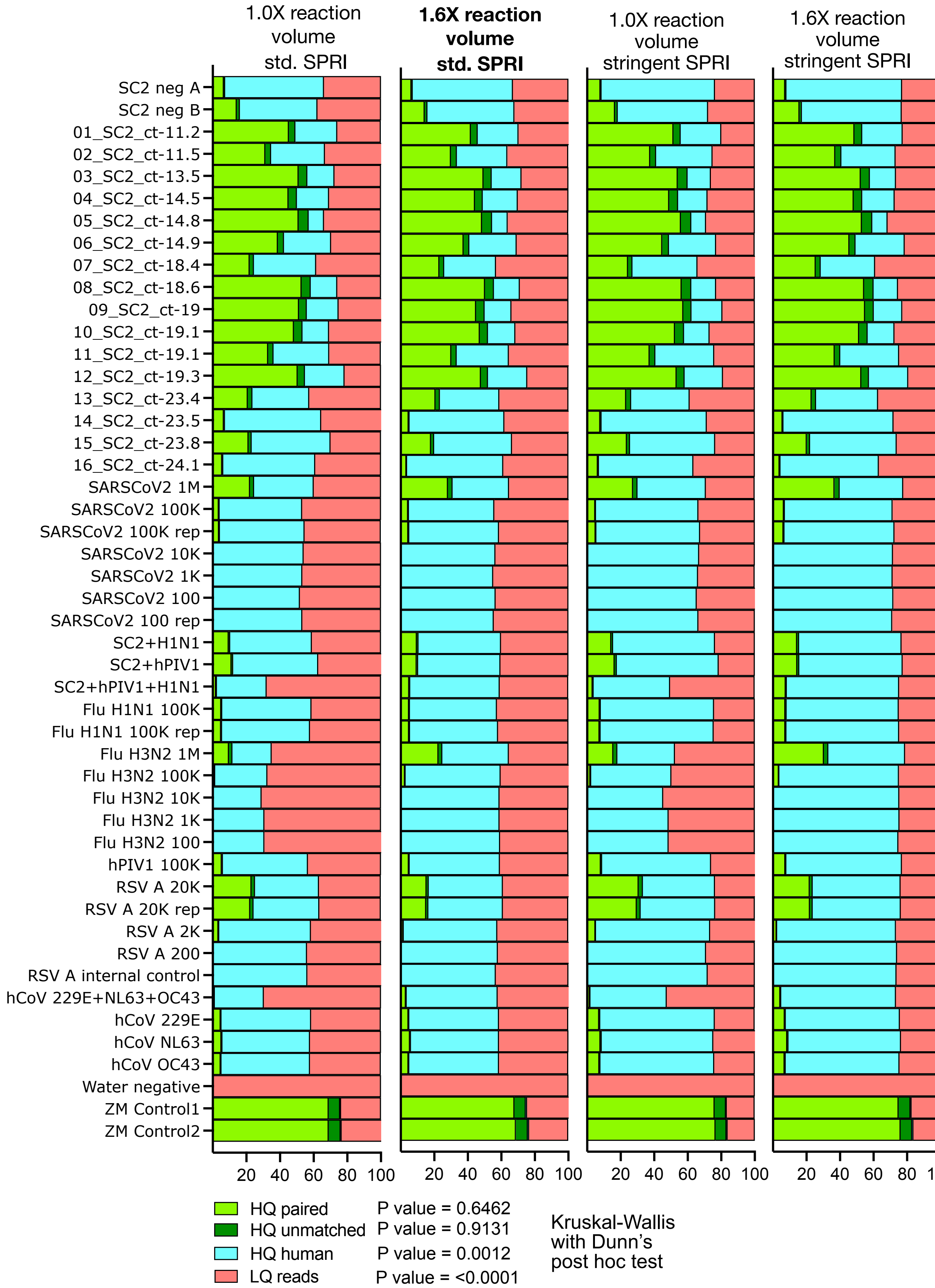

Total R1R2 raw reads

1X std SPRI	1.6X std SPRI	1X stringent SPRI	1.6X stringent SPRI
733,994	857,950	616,614	785,644
417,190	589,396	332,080	546,088
21,920,762	20,991,210	20,953,826	17,811,910
4,098,086	4,013,338	3,309,066	2,956,686
49,173,760	54,891,978	51,715,602	58,020,726
20,847,324	22,301,674	21,396,678	22,875,250
7,187,558	7,223,668	6,886,686	7,087,758
6,824,096	9,727,292	6,276,044	8,644,510
852,538	1,290,798	816,968	1,217,728
10,019,212	7,792,800	10,539,848	8,142,898
4,061,606	2,900,622	4,044,990	2,380,206
2,470,680	2,210,864	2,431,804	2,194,692
1,169,886	1,467,432	1,113,904	1,307,178
22,624,310	21,108,918	23,265,814	20,021,278
851,704	906,186	790,278	823,300
545,036	590,562	513,582	537,160
858,332	779,988	824,394	717,942
484,020	607,256	486,730	578,308
11,186,916	12,807,338	9,099,152	10,853,410
6,010,502	7,073,224	4,457,474	5,269,478
4,877,526	6,695,092	3,698,132	5,513,100
4,892,318	6,519,330	3,690,192	5,107,078
4,760,794	6,412,814	3,571,298	4,800,980
5,784,786	3,179,048	4,215,880	2,485,862
5,060,408	7,641,680	3,768,066	5,880,482
6,426,966	4,642,826	4,860,372	3,546,774
4,422,504	5,910,650	3,314,684	4,520,178
6,597,262	6,115,214	3,051,350	4,859,784
5,618,580	5,019,562	4,258,334	3,774,318
5,214,364	4,165,960	3,772,944	3,098,320
8,864,516	10,746,358	4,223,802	9,275,358
5,707,040	5,390,902	2,719,000	4,266,998
3,654,658	5,700,870	1,651,046	4,509,478
4,283,330	5,253,630	1,898,664	4,056,422
5,059,702	4,772,158	2,204,986	3,714,870
5,134,582	4,533,498	3,711,244	3,448,956
10,336,738	8,633,824	8,471,112	7,243,282
13,283,660	10,389,990	11,061,226	8,275,882
7,283,690	6,355,618	5,601,622	5,055,048
6,582,466	5,567,198	4,890,132	4,337,478
5,081,708	6,395,388	3,587,004	4,932,622
4,736,064	5,478,760	2,097,688	4,218,808
4,549,486	5,464,904	3,309,672	4,270,624
5,949,922	4,041,634	4,397,724	3,055,998
4,787,186	3,556,966	3,438,776	2,716,432
288	284	74	118
6,254,674	6,547,888	5,926,162	6,685,070
914,514	937,882	843,102	923,916

Fig. S6: Relative frequencies of HQ paired end, unmatched reads, human reads and low-quality reads across the four different SPRI methods (Pilot 3)

Kneaddata filtered reads with a minimum length of 70 bp were plotted for HQ paired end reads, HQ unmatched reads, human reads and LQ reads for all four laboratory SPRI methods. The number of total R1 and R2 raw reads is shown in the table. The Kruskal-Wallis test with Dunn's multiple comparisons was employed to identify statistical differences between the four distinct laboratory methods for each of the read groups. Abbreviations: SC2 = SARS-CoV-2; ct = PCR cycle threshold for the detection of SARS-CoV-2; rep = replicate sequencing; 1M = 1 million copy number sample; 100k, 10k, 1k, = 100,000, 10,000 and 1000 copy number sample, 100 = 100 copy number sample. SPRI stands for solid phase reversible immobilisation. Two different SPRI conditions were tested – standard and stringent. Two different cDNA synthesis reaction volumes were tested, at 1X and 1.6X scale.
