# Supplementary material for: Benchmarking and optimisation of bait-capture metagenomics for sequencing of respiratory viruses at scale
Source: Genome Med. 2026 Jul 30;18:166. doi: 10.1186/s13073-026-01725-8 (PMC13715170; doi:10.1186/s13073-026-01725-8)
Supplement: Supplementary file 11 [file 13073_2026_1725_MOESM9_ESM.pdf]

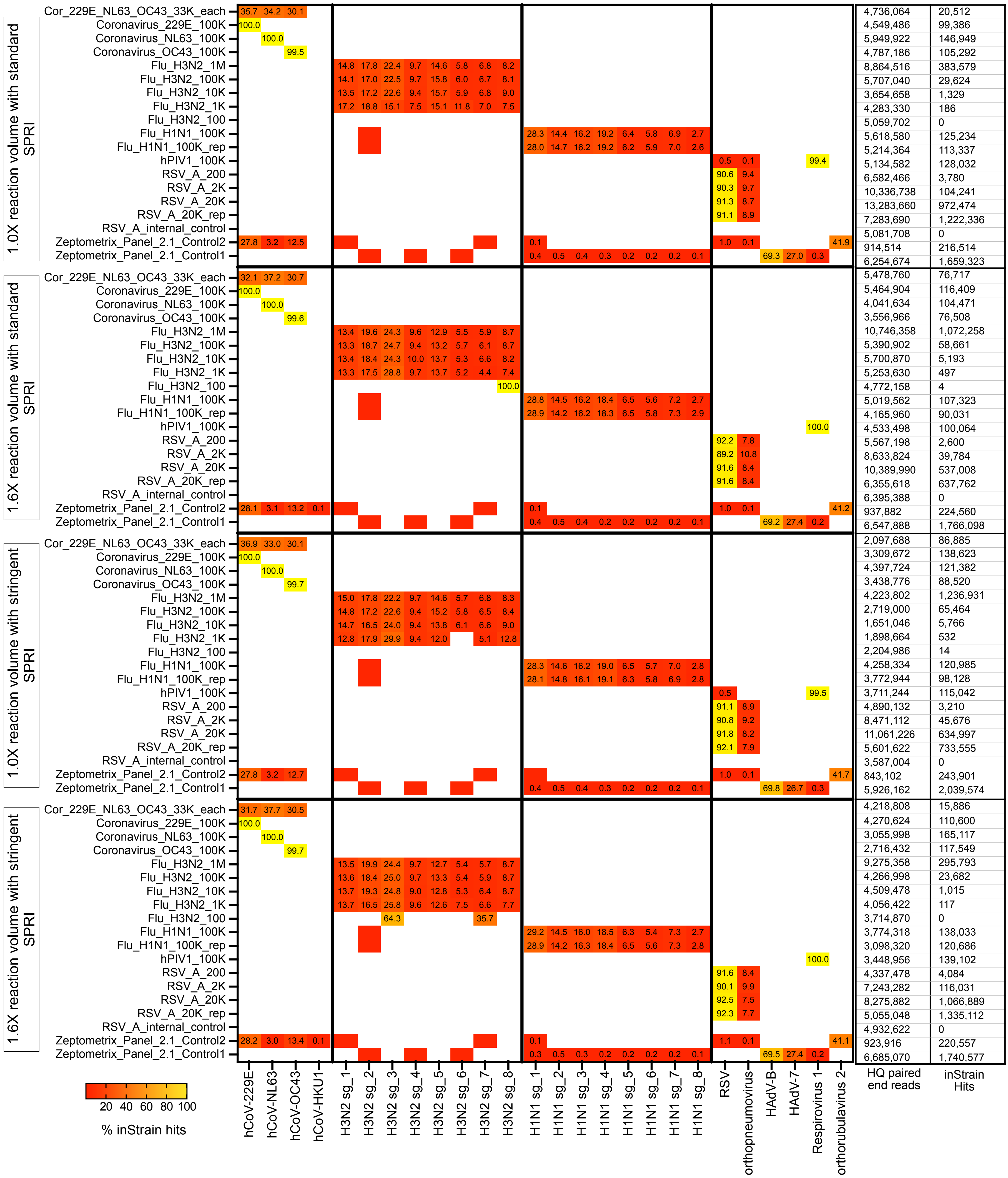

Fig. S7: inStrain classification for hCoV, Flu H3N2 and H1N1, HPIV1, RSV A and ZeptoMetrix control samples

High-quality (HQ) paired-end reads with a minimum length of 70 bp were classified using inStrain with the proportional abundances presented in a heat map with one decimal value. The absence of a label in a coloured heatmap cell indicates that the value is less than 0.1. The table situated to the right of the heatmap displays the total number of HQ R1R2 paired-end reads used for classification, along with the number of inStrain hits. Abbreviations: SPRI: solid phase reversible immobilisation, 1M = one million copies, k = thousand, sg_1 to sg_8 = individual H3N2 and H1N1 segments 1 to 8. It should be noted that human parainfluenza virus 1 has been classified as Respirovirus 1, HAdV = human adenovirus, and H orthorubulavirus = human parainfluenza virus 2
